# Pathogenesis of FOLFOX induced sinusoidal obstruction syndrome in a murine chemotherapy model

**DOI:** 10.1016/j.jhep.2013.04.014

**Published:** 2013-08

**Authors:** S.M. Robinson, J. Mann, A. Vasilaki, J. Mathers, A.D. Burt, F. Oakley, S.A. White, D.A. Mann

**Affiliations:** 1 Institute of Cellular Medicine, Newcastle University, Framlington Place, Newcastle upon Tyne NE2 4HH, UK; 2 Department of HPB Surgery, Freeman Hospital, High Heaton, Newcastle upon Tyne NE7 7DN, UK; 3 Institute of Ageing and Chronic Disease, University of Liverpool, Daulby Street, Liverpool L69 3GA, UK; 4 Human Nutrition Research Centre, Centre for Brain Ageing and Vitality, Institute for Ageing and Health, Newcastle University, Campus for Ageing and Vitality, Newcastle upon Tyne NE4 5PL, UK; 5 School of Medicine, The University of Adelaide, Frome Road, Adelaide 5005, Australia

**Keywords:** AST, aspartate aminotransferase, ALT, alanine aminotransferase, ALP, alkaline phosphatase, BHA, butylated hydroxyanisole, CRLM, colorectal liver metastases, CXCL1/2, chemokine (C-X-C motif) ligand 1/2, GAPDH, gyceraldehyde 3-phosphate dehydrogenase, H&E, haematoxylin and eosin, HPF, high powered field, γH2AX, phosphorylated form of the H2A histone family, member X, i.p., intraperitoneal, IL-6, interleukin 6, MCP1, monocyte chemotactic protein-1, NAC, N-acetylcysteine, NRF2, nuclear factor (erythroid-derived 2)-like 2, NQO1, NAD(P)H dehydrogenase 1, PCNA, proliferating cell nuclear antigen, PAI-1, plasminogen activatior inhibitor 1, PAR 1/2, protease activated receptor 1/2, STAT3, signal transducer and activator of transcription 3, SOS, sinusoidal obstruction syndrome, TXN1, thioredoxin 1, VEGF-A/B/C, vascular endothelial growth factor A/B/C, VEGFR-1/2, vascular endothelial growth factor receptor ½, vWF, von Willebrand factor, Sinusoidal obstruction syndrome, Oxaliplatin, Colorectal liver metastases, Chemotherapy induced liver injury

## Abstract

**Background & Aims:**

Sinusoidal obstruction syndrome (SOS) following oxaliplatin based chemotherapy can have a significant impact on post-operative outcome following resection of colorectal liver metastases. To date no relevant experimental models of oxaliplatin induced SOS have been described. The aim of this project was to establish a rodent model which could be utilised to investigate mechanisms underlying SOS to aid the development of therapeutic strategies.

**Methods:**

C57Bl/6 mice, maintained on a purified diet, were treated with intra-peritoneal FOLFOX (n = 10), or vehicle (n = 10), weekly for five weeks and culled one week following final treatment. Sections of the liver and spleen were fixed in formalin and paraffin embedded for histological analysis. The role of oxidative stress on experimental-induced SOS was determined by dietary supplementation with butylated hydroxyanisole and N-acetylcysteine.

**Results:**

FOLFOX treatment was associated with the development of sinusoidal dilatation and hepatocyte atrophy on H&E stained sections of the liver in keeping with SOS. Immunohistochemistry for p21 demonstrated the presence of replicative senescence within the sinusoidal endothelium.

FOLFOX induced endothelial damage leads to a pro-thrombotic state within the liver associated with upregulation of PAI-1 (*p* <0.001), vWF (*p* <0.01) and Factor X (*p* <0.001), which may contribute to the propagation of liver injury.

Dietary supplementation with the antioxidant BHA prevented the development of significant SOS.

**Conclusions:**

We have developed the first reproducible model of chemotherapy induced SOS that reflects the pathogenesis of this disease in patients. It appears that the use of antioxidants alongside oxaliplatin based chemotherapy may be of value in preventing the development of SOS in patients with colorectal liver metastases.

## Introduction

A significant proportion of patients presenting with colorectal liver metastases (CRLM) have a pattern of disease that often prevents safe surgical resection. For these patients, the treatment of choice is systemic chemotherapy in order to downstage disease in preparation for curative surgery. The most widely utilised regimens for this purpose consist of a thymidylate synthase inhibitor (either 5-FU/folinic acid or capecitabine) in combination with either oxaliplatin or irinotecan and, more recently, with the addition of antibody therapy in the form of bevacizumab or cetuximab [1], [2]. The success of these regimens in achieving effective tumour downstaging results in increasing numbers of patients undergoing liver resection who have received multiple cycles of prolonged chemotherapy prior to curative surgery.

Oxaliplatin based regimens have been associated with the development of injury to the hepatic parenchyma in the form of sinusoidal obstruction syndrome (SOS) [3]. SOS is characterised by hepatic sinusoidal dilatation, hepatocyte atrophy, peri-sinusoidal fibrosis, and nodular regenerative hyperplasia [3]. These histological changes appear to be present in up to 40% of patients treated with oxaliplatin based regimens undergoing liver resection [4], [5], [6], [7], [8]. The presence of SOS is a cause for concern when undertaking major hepatectomy as it is associated with increased peri-operative morbidity including post-hepatectomy liver failure characterised by cholestasis, ascites, and a prolonged prothrombin time [9], [10]. More recently, it has been demonstrated that the presence of SOS is associated with poorer long-term disease specific survival following resection of CRLM and, in particular, an increased risk of intrahepatic recurrence [6], [11].

At present very little is known about the pathophysiological mechanisms which lead to the development of oxaliplatin-induced SOS and research on these mechanisms is limited by the lack of an appropriate and reproducible experimental model of oxaliplatin induced SOS. Whilst the administration of monocrotaline to rats leads to similar histological lesions within the liver, it is not clear if the mechanisms responsible for these lesions are shared by oxaliplatin.

The aim of the current study was to establish a model of oxaliplatin induced SOS relevant to the use of conversion chemotherapy and to identify molecular mechanisms involved in its pathogenesis.

## Materials and methods

The use of animals in this study was reviewed and approved by the local research ethics committee and the UK home office.

### *In vivo* chemotherapy model

To determine the direct toxicity of FOLFOX, 10-week old C57Bl/6 mice were treated with intraperitoneal (i.p.) oxaliplatin 6 mkg/kg followed 2 h later by 5-FU 50 mg/kg and folinic acid 90 mg/kg (all obtained from Sigma-Aldrich, Dorset, UK), on a weekly basis for 5 weeks. The drug dosing schedule was based upon that used in previously published studies and our own preliminary dose finding experiments [12], [13], [14]. Control animals received vehicle alone. There were 10 animals per treatment group, although one animal in each group died during the course of the experiment. Mice were culled one week after the final dose of chemotherapy under isoflurane anaesthesia by cardiac puncture. All animals received standard animal house care, including *ad libitum* access to water and a standard purified diet (D01060501, Research Diets Inc, New Brunswick, USA).

To determine the effect of antioxidant therapy on the development of FOLFOX induced SOS, the above experiments were repeated (n = 5 per group) with custom diets supplemented with either 3% N-acetylcysteine or 0.7% butylated hydroxyanisole (Research Diets Inc, New Brunswick, USA). Based upon a typical dietary intake of 150 g food per kg body weight, this equated to a daily dose of 4.5 g/kg NAC and 1 g/kg BHA per day. The diets were otherwise identical to the standard purified diet used above. There was one death in the FOLFOX treated group receiving a BHA supplemented diet.

### Microarray analysis

An Illumina mouse microarray (Illumina Inc, San Diego, USA) was performed on mRNA isolated from the livers of 6 vehicle treated animals and 6 FOLFOX treated animals (service provided by Genome Centre, Queen Mary University of London, UK). Analysis was performed using GeneSpring GX software (Agilent Technologies Inc, Santa Clara, USA).

### Quantitative RT-PCR

Whole liver RNA was isolated using the RNeasy system (Qiagen, West Sussex, UK) according to the manufacturers’ protocol. cDNA was generated using 1 μg RNA, MMLV-RT, and a random hexamer primer (Promega, Southampton, UK). SYBR-green reagents (Sigma-Aldrich, Dorset, UK) were utilised for qRT-PCR with fold change in gene expression calculated using the ΔΔCT method. *GAPDH* was used as the internal control. Primer sequences are listed in Supplementary Table 1.

### Western blot

Protein was extracted from whole liver tissue in RIPA buffer and quantified using the Bradford assay (Bio Rad, UK). Fifty μg of protein was loaded onto acrylamide gels and separated by SDS-page prior to transfer to nitrocellulose. Proteins were detected with antibodies as listed in Supplementary Table 2 and visualised by chemiluminescence. GAPDH was used as the loading control.

### Total glutathione content

The automated glutathione recycling method described by Anderson was used to assess the total glutathione content in liver samples [15], using a 96-well plate reader (Powerwave X340, Bio-tech instruments Inc, Vermont, USA). The protein content of samples was determined using the method of Lowry *et al.* [16].

### ELISA

Serum was separated from clotted whole blood samples obtained after cardiac puncture and diluted 1:3 in PBS/1% BSA. CXCL1 and VEGF were measured in the serum using a commercial ELISA development kit, according to the manufacturers’ instructions (R&D Systems, Minneapolis, USA).

### Histological assessment

Five μm thick slices of tissue were cut from formalin fixed and paraffin embedded tissue and stained with haematoxylin and eosin (H&E). The presence of sinusoidal injury was assessed by two specialist liver pathologists blinded to the treatment group (ADB and FO). In addition, the degree of sinusoidal dilatation was graded according to the method of Rubbia-Brandt. The disruption and loss of the endothelial layer within sinusoids were graded as either absent or present [17].

### Immunohistochemistry

Sections were deparaffinised, rehydrated and endogenous peroxidase activity blocked. Non-specific antibody binding was prevented by blocking of avidin and biotin (Vector Laboratories, Burlingame, USA) followed by incubation for 30 min in 20% swine serum. Sections were incubated with the primary antibody overnight at 4 °C. The next morning, sections were incubated with a biotin conjugated secondary antibody for 2 h followed by a 1-h incubation with streptavidin biotin-peroxidase complex (Vector Laboratories, Burlingame, USA). Staining was visualised with 3,3′-diaminobenzidine tetrahydrochloride (Vecto Laboratories, Burlingame, USA) and sections counterstained with haematoxylin. The details of the antibodies used along with methods of antigen retrieval are listed in Supplementary Table 3. Staining was quantified using Leica Qwin software (Leica Microsystem (UK) Ltd, Buckinghamshire, UK) using 20× magnification with 25 images per slide being assessed.

Splenic megakaryocytes were assessed in 10× magnified sections with results representing the mean count for 15 images per section.

### Statistical analysis

Continuous variables were assessed with Mann-Whitney *U*-Test with *p* <0.05 considered statistically significant. Categorical variables were compared with Fisher’s exact test. Analysis was performed in GraphPad Prism (GraphPad Software, La Jolla, USA). On figures, *p* values are abbreviated as ^∗^⩽0.05, ^∗∗^⩽0.01, ^∗∗∗^⩽0.001.

## Results

### Development of a reproducible *in vivo* experimental model

**Fig. 1:**
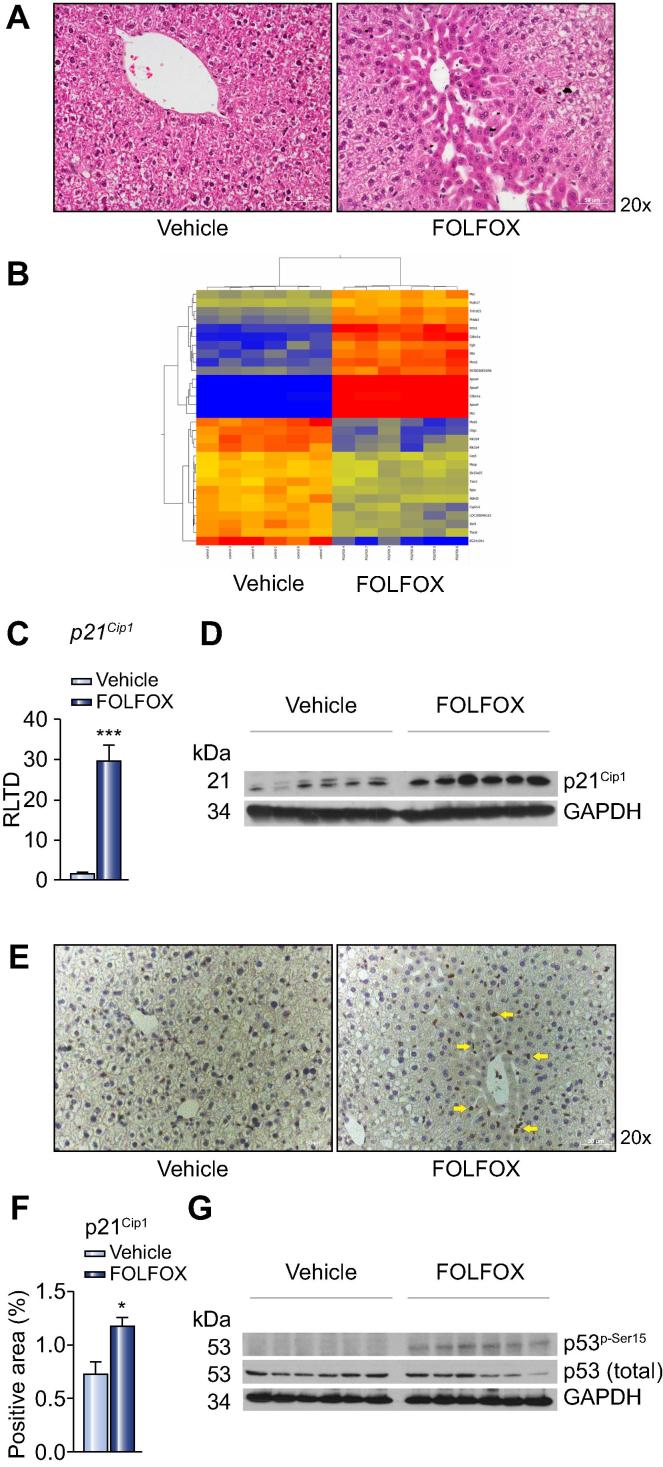
**Endothelial senescence in FOLFOX induced SOS**. (A) Mice maintained on a purified diet and treated with intraperitoneal FOLFOX for 5 weeks (n = 9 per group) demonstrate histological changes of SOS on H&E stained sections of the liver. (B) To identify potential biological processes implicated in the pathogenesis of FOLFOX induced SOS, a microarray was performed using mRNA isolated from 6 animals in each group, a heat map of which is shown. (B) Cellular senescence was identified as a key mechanism in FOLFOX induced SOS, with upregulation of p21^Cip1^ at both a transcript level (C; n = 8 animals per group) and protein level (D; representative blot of n = 6 per group). (E) Immunohistochemistry revealed that p21Cip1 staining is limited to endothelial cells at the site of sinusoidal injury (yellow arrows). (F) Densitometry (n = 9 animals per group) confirmed an increased intensity of p21^Cip1^ staining. (G) It is likely that endothelial senescence is driven by p53 with Western blot of whole liver protein extracts revealing increased phosphorylation of this transcription factor at serine 15 (representative blot of n = 6 per group). ^∗^*p* <0.05; ^∗∗∗^*p* <0.001. (This figure appears in colour on the web.)

To assess the effect of FOLFOX on liver parenchyma, C57Bl/6 mice were treated with i.p. FOLFOX for 5 weeks and culled one week after the final dose. Blinded review of H&E stained sections of the liver demonstrated evidence of sinusoidal dilatation and associated hepatocyte atrophy (Fig. 1A) in all 9 FOLFOX treated animals, whereas they were absent in all animals in the control arm (*p* <0.0001). These histological changes were present homogenously throughout the liver with sinusoidal dilatation being of grade 1 in all animals in the FOLFOX group, whereas there was no sinusoidal dilatation detected in any animal in the control group.

Similarly, sinusoidal endothelial disruption was absent within all animals in the control arm, but was present in every FOLFOX treated animal (*p* <0.0001; Supplementary Table 4).

The presence of SOS was associated with evidence of hepatocellular injury as demonstrated by elevated AST and ALT in FOLFOX treated animals (Supplementary Fig. 1A). It is noteworthy that when these experiments were performed utilising a standard chow diet, no such histological changes were demonstrated (Supplementary Fig. 1B). This model has been repeated on 3 separate occasions and the histological changes found to be entirely reproducible with minimal variation in the severity of injury.

In order to identify the key molecular mechanisms underpinning the development of SOS following FOLFOX administration, a microarray was performed on whole liver mRNA from 6 animals in each group. This demonstrated upregulation greater than 1.5 fold of 338 transcripts and downregulation greater than 1.5 fold of 267 transcripts, in FOLFOX treated animals (Fig. 1B; Supplementary Table 5). These results were validated by performing qRT-PCR for the 7 most highly up and downregulated transcripts (Supplementary Table 6). Pathway analysis using GeneSpring GX software revealed that biological pathways related to cell cycle regulation, oxidative stress, angiogenesis, matrix remodelling, and the coagulation cascade may all be implicated in the pathogenesis of FOLFOX induced SOS and these were therefore explored in greater detail. The key genes identified from the microarray for each of these pathways are summarised in Supplementary Table 7.

### Cell cycle regulation

Microarray analysis of liver tissue revealed one of the most highly upregulated genes in FOLFOX treated animals to be the cyclin dependent kinase inhibitor *p21*^Cip1^. This finding was confirmed both at the mRNA level by qRT-PCR (21 fold; *p* <0.001; Fig. 1C) and protein level by Western blot (Fig. 1D). Immunohistochemistry demonstrated a 1.7 fold increase in *p21*^Cip1^ expression (*p* <0.05), which was localised predominantly to endothelial cells in areas of sinusoidal injury (Fig. 1E and F).

Increased p21^Cip1^ expression is classically considered to be a marker of replicative senescence, a process which is characterised by cell cycle arrest in G1 phase [18]. Oxaliplatin induced SOS has previously been associated with upregulation of the tissue plasminogen activation inhibitor PAI-1 (SERPINE1), a finding which is consistent with replicative senescence in this condition and which has been confirmed in the current model with a 2800 fold increase at the mRNA level in the liver of FOLFOX treated mice (*p* <0.001; Supplementary Fig. 2A) [19], [20]. Immunohistochemistry for the senescence marker γH2AX demonstrated a significant increase in the number of positive cells within the liver of FOLFOX treated mice, the pattern of staining of which was similar to that of p21^Cip1^ (*p* <0.001; Supplementary Fig. 2B and C).

The expression of both p21^Cip1^ and PAI-1 is regulated by the transcription factor p53 [21]. In response to cellular injury, e.g., DNA damage or oxidative stress, p53 undergoes phosphorylation and becomes transcriptionally active [22]. In mice with FOLFOX induced SOS, increased phosphorylation of p53 at serine 15 was detected in whole liver protein extracts (Fig. 1G), suggesting that this may be a driver of senescence in this context.

**Fig. 2:**
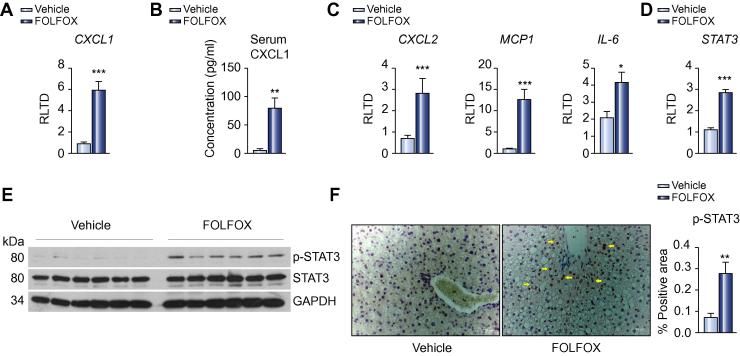
**Cytokine response to sinusoidal injury**. (A) Microarray suggested upregulation of the senescence associated chemokine *CXCL1* in the liver of FOLFOX treated mice, which was confirmed by qRT-PCR (n = 8 animals per group). (B) Furthermore, CXCL1 was demonstrated to be elevated within the serum of mice with FOLFOX induced SOS (n = 9 animals per group). (C) qRT-PCR demonstrated upregulation of other senescence associated inflammatory mediators including *CXCL2*, *MCP*-1, and *IL-6* (n = 8 animals per group). (D) IL-6 is more typically described as a pro-proliferative cytokine implicated in liver regeneration where it signals through activation of the transcription factor STAT3. Microarray and subsequent qRT-PCR demonstrated increased expression of this transcription factor in FOLFOX treated animals (n = 8 animals per group). Western blot demonstrated activation of this transcription factor by phosphorylation (E; representative blot of n = 6 per group), which immunohistochemistry revealed to be occurring predominantly in hepatocytes around portal tracts (F). Densitometry confirmed the increase in phosphorylated STAT3 seen on Western blot (F; n = 9 animals per group). ^∗^*p* <0.05; ^∗∗^*p* <0.01; ^∗∗∗^*p* <0.001. (This figure appears in colour on the web.)

Cellular senescence is associated with increased expression of a variety of chemokines/cytokines. Microarray analysis revealed that one such chemokine, CXCL1, was upregulated 3.3 fold in the liver of FOLFOX treated animals as compared to controls, a finding confirmed by qRT-PCR (Fig. 2A; *p* <0.001). CXCL1 is the murine homologue of the human cytokine IL-8, increased levels of which have been reported in the serum of patients with SOS following bone marrow transplantation [23]. In keeping with this, there was a dramatic increase in the serum CXCL1 concentration of FOLFOX treated mice (Fig. 2B; *p* <0.01).

qRT-PCR was able to confirm upregulation of other key inflammatory mediators implicated in the senescence response (Fig. 2C) including CXCL2 (4.2 fold; *p* <0.001), MCP-1 (12.5 fold; *p* <0.001) and IL-6 (2.0 fold; *p* <0.05) in the liver of FOLFOX treated mice. Whilst IL-6 has been implicated in the maintenance of senescence [24], it is more widely considered as a pro-proliferative cytokine, for example in liver regeneration, where it signals through JAK1/STAT3 [25]. Micro-array, and subsequent qRT-PCR, demonstrated that hepatic expression of STAT3 was significantly increased in the liver of FOLFOX treated mice (Fig. 2D; *p* <0.001), suggesting a potential role for this pathway in the pathogenesis of SOS and, therefore, this was explored in more detail.

In response to IL-6, STAT3 is activated by phosphorylation, whereby it acts as a transcriptional regulator of a wide variety of target genes which, in the liver, are involved in a variety of processes, including hepatocyte regeneration [26]. Western blot demonstrated increased phosphorylation of STAT3 within the liver of FOLFOX treated mice (Fig. 2E), which immunohistochemistry demonstrated to be located predominantly within the nucleus of hepatocytes around portal tracts (Fig. 2F). It is of note that this is the region in which regenerative hyperplasia is seen in patients with advanced SOS and, as such, it appears likely that this is driven by IL-6 mediated activation of STAT3. In support of this hypothesis, there was a 1.8-fold increase in density of PCNA staining in the liver of animals with SOS (*p* <0.05; Supplementary Fig. 3).

### Oxidative stress induced sinusoidal injury

One of the mechanisms for cellular excretion of oxaliplatin is through conjugation of the active drug to glutathione, which can then lead to intracellular depletion of this antioxidant [27]. It is known that endothelial depletion of glutathione is a key process in monocrotaline induced SOS [28]. Compared with animals treated with vehicle control alone, total liver glutathione in mice with FOLFOX induced SOS was significantly reduced (95.4 *vs.* 76.9 μmoles/g protein; *p* <0.05; Supplementary Fig. 4A). In support of a role for oxidative stress in the pathogenesis of oxaliplatin induced SOS, microarray revealed upregulation of a variety of genes implicated in the response to oxidative stress, including metallothionein 1 (*Mt1*), heme oxygenase 1 (*HO1*), and superoxide dismutase 3 (*SOD3*), the expression of all of which was confirmed by qRT-PCR (Supplementary Fig. 4B) and, in the case of SOD3, by Western blot (Supplementary Fig. 4C).

**Fig. 3:**
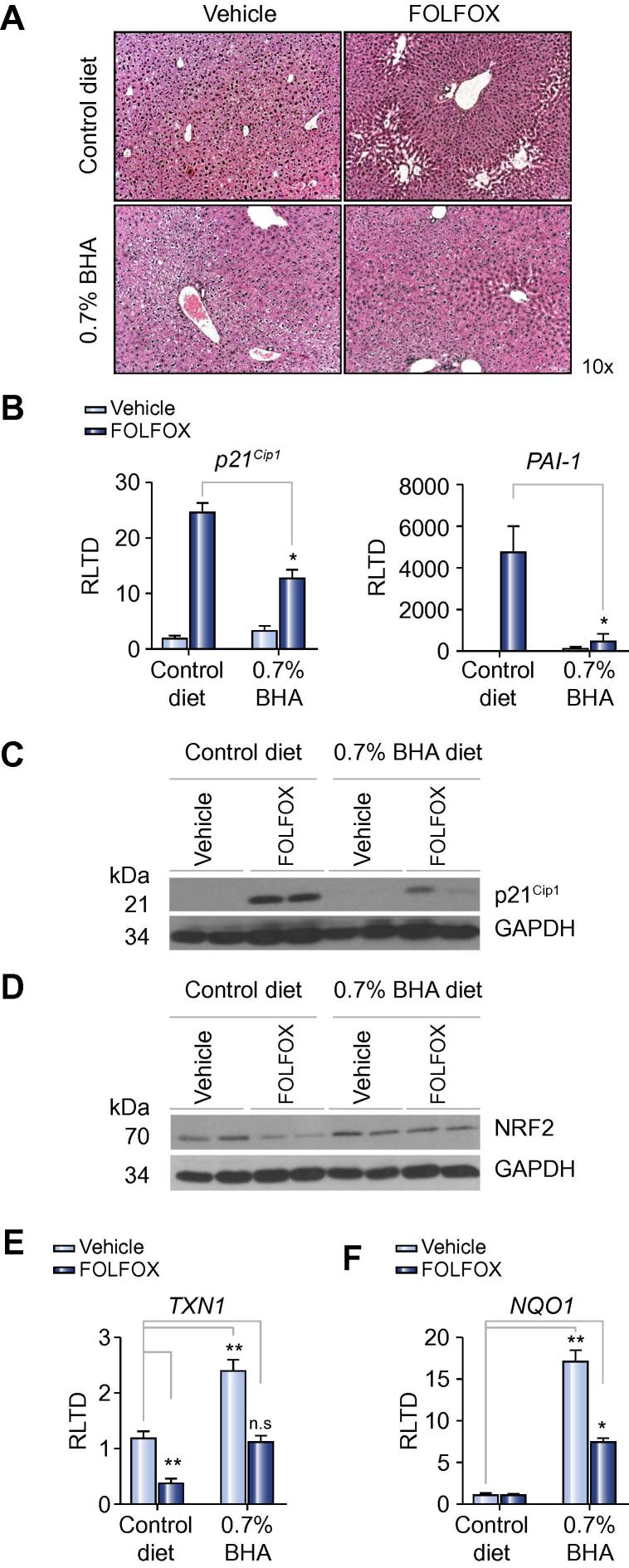
**Oxidative stress in FOLFOX induced SOS**. (A) To assess the role of oxidative stress in the development of SOS, the experiment was repeated, but this time supplementing the diet with 0.7% BHA with the purified diet serving as a control. Supplementation with 0.7% BHA prevented the development of sinusoidal injury. (B) In keeping with this, there was decreased expression of *p21*^Cip1^ and *PAI-1* transcript (n = 5 per group, n = 4 in the FOLFOX and BHA group) in the livers of these animals. (C) The reduction in p21 expression was confirmed at a protein level by Western blot (representative blot of n = 2 per group). (D) FOLFOX induced SOS is associated with diminished expression of the antioxidant transcription factor NRF2 which is prevented when BHA is administered (representative blot of n = 2 per group). (E) BHA supplementation is associated with increased expression of the NRF2-regulated antioxidant genes *TXN1* and *NQO1* (n = 5 per group, n = 4 in the FOLFOX and BHA group). ^∗^*p* <0.05; ^∗∗^*p* <0.01; n.s, not significant. (This figure appears in colour on the web.)

To determine if antioxidant therapy could be of value in preventing the development of oxaliplatin induced SOS, the current experiment was repeated utilising dietary supplementation with either 3% N-acetylcysteine (NAC) or 0.7% butylated hydroxyanisole (BHA), both of which are effective anti-oxidants in mice [29], [30]. The standard purified diet was used as a control. Dietary supplementation was commenced 1 week prior to starting FOLFOX and continued until animals were culled one week after the final dose. Review of H&E stained sections showed that NAC treatment had no effect on SOS development whereas BHA reduced the severity of sinusoidal injury (Fig. 3A; Supplementary Fig. 4D). Specifically, the incidence of endothelial disruption was reduced to only 25% in FOLFOX treated animals receiving a BHA supplemented diet (*p* <0.05; Supplementary Table 8).

In keeping with the histological findings, there was a statistically significant reduction in the senescence markers p21^Cip1^ and PAI-1 at the transcript level, in the liver of FOLFOX treated mice receiving the BHA supplemented diet as compared with control diet (Fig. 3B). The reduction in p21^Cip1^ expression in mice receiving BHA supplementation was confirmed by Western blot (Fig. 3C).

BHA exerts its effect through increased transcriptional activity of the antioxidant transcription factor NRF2 [30]. Relative to control animals, hepatic NRF2 protein expression levels were reduced in mice treated with FOLFOX (Fig. 3D). This diminution of NRF2 was prevented in FOLFOX treated animals receiving the BHA supplemented diet (Fig. 3D). To determine the functional effects of BHA on transcription of genes downstream of NRF2, qRT-PCR was performed for the classically NRF2 regulated antioxidant genes *TXN1* (thioredoxin 1) and *NQO*1 (NAD(P)H dehydrogenase 1). In animals with FOLFOX induced SOS, we observed a 3-fold reduction in TXN1 expression (*p* <0.01; Fig. 2E) which was prevented in animals receiving BHA supplementation. SOS was not associated with a reduction in NQO1 expression, however, BHA supplementation was associated with a 19-fold increase in NQO1 expression (*p* <0.01; Fig. 2F) suggesting added protection from oxidative stress in these animals.

### Angiogenesis in FOLFOX induced SOS

**Fig. 4:**
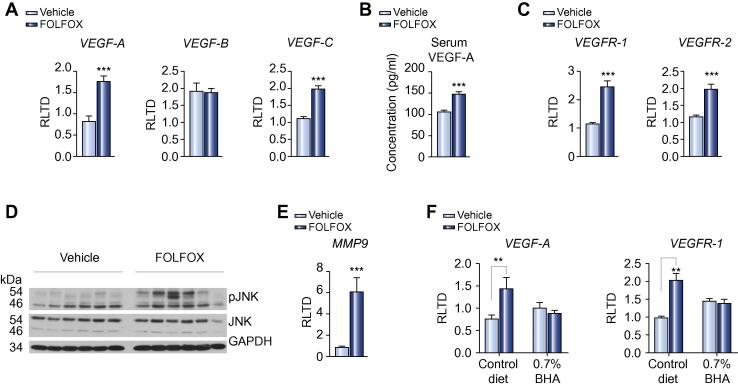
**Angiogenesis in FOLFOX induced SOS**. (A) FOLFOX induced SOS is associated with upregulation of the angiogenic factors *VEGF-A* and *VEGF-C*, but not *VEGF-B*, at the transcript level within the liver (n = 8 per group). (B) This is reflected in an increase in serum VEGF-A levels in mice with FOLFOX induced SOS (n = 9 per group). (C) In addition there is increased expression of the VEGF receptors VEGFR-1 and VEGFR-2 within the liver of FOLFOX treated animals (n = 8 per group). Previous reports have suggested that, in the context of SOS, VEGF mediated signalling results in phosphorylation of JNK leading to increased expression of MMP-9 [31]. In support of a role for this mechanism in FOLFOX induced SOS, (D) Western blot revealed increased phosphorylation of JNK within the liver of FOLFOX treated animals (representative blot of n = 6 per group) with (E) an associated increase in *MMP-9* transcript (n = 8 per group). (F) BHA treatment protects against the FOLFOX induced increase in both *VEGF-A* and *VEGFR-1* transcript expression (n = 5 per group, n = 4 in the FOLFOX and BHA group). ^∗∗^*p* <0.01; ^∗∗∗^*p* <0.001.

There is a growing body of evidence from several cohort studies to suggest that the addition of bevacizumab, a monoclonal antibody directed against VEGF-A, to oxaliplatin based chemotherapy can reduce the incidence of SOS by approximately a third in patients [7]. In support of these observations, we were able to demonstrate increased transcript expression of the angiogenesis related genes *VEGF-A* (2.1 fold; *p* <0.001) and *VEGF-C* (1.8 fold; *p* <0.001), but not *VEGF-B*, in mice with FOLFOX induced SOS (Fig. 4A). This was reflected in an increased serum concentration of VEGF-A in FOLFOX treated mice (*p* <0.001; Fig. 4B).

The microarray study demonstrated upregulation of members of the VEGF family with subsequent qRT-PCR, demonstrating a 2 fold increase in expression of both VEGFR-1 and VEGFR-2 (Fig. 4C). It has recently been reported in the monocrotaline model that signalling through the VEGF system leads to phosphorylation of c-Jun N-terminal kinase (JNK) and subsequent upregulation of MMP9 expression, which plays a pivotal role in the pathogenesis of SOS [31]. This mechanism also appears to be of relevance in oxaliplatin induced SOS, with Western blot demonstrating increased JNK phosphorylation and *MMP9* transcript expression within the liver of FOLFOX treated animals (Fig. 4D and E). It is also noteworthy that dietary supplementation with 0.7% BHA prevented the FOLFOX induced increase in *VEGF-A* and *VEGFR-1* transcripts, suggesting that the level of expression correlates with the severity of liver injury (Fig. 4F).

The effects of FOLFOX on the VEGF system appeared to be specific with no evidence of increased expression of other key angiogenic factors (i.e., angiopoietin 1 and 2) or their receptors (i.e., TIE 2) being detected within the liver of FOLFOX treated animals (data not shown).

### SOS is associated with a pro-thrombotic environment within the injured hepatic sinusoid

Analysis of the microarray suggested activation of the coagulation cascade in the pathogenesis of oxaliplatin induced SOS, with upregulation of von Willebrand factor (vWF), a key component in platelet adhesion, in the liver of FOLFOX treated mice, which was confirmed by qRT-PCR (*p* <0.01; Supplementary Fig. 5A). Given the observation that splenomegaly is a feature of patients treated with oxaliplatin induced SOS [32], we reviewed H&E stained sections of the spleen of FOLFOX treated mice and observed the presence of large clusters of megakaryocytes within these animals, which were not present within control animals (*p* <0.001; Supplementary Fig. 5B and C). This suggests that an increasing number of platelets may be released directly into the portal circulation of FOLFOX treated animals which, in the context of the elevated liver vWF expression, may be of importance in the pathogenesis of SOS.

Further support for this hypothesis is that a pro-thrombotic factor is of importance in the pathogenesis of oxaliplatin induced SOS, such that Factor X transcript expression is upregulated within the liver of FOLFOX treated mice (*p* <0.001; Supplementary Fig. 5D). Additionally increased expression of tissue factor, an essential component in the activation of Factor X, was observed by immunohistochemistry in the region of injured hepatic sinusoids (Supplementary Fig. 5E). In keeping with a role for this process in driving the matrix remodelling associated with SOS, FOLFOX treatment was also associated with increased hepatic expression of the Factor Xa receptors PAR1 (3.9 fold; *p* <0.01) and PAR2 (2.1 fold; *p* <0.05) (Supplementary Fig. 5F).

The effects of FOLFOX treatment on the coagulation system appear to be independent of oxidative stress, with 0.7% BHA treatment having no effect on the expression of either Factor X or vWF (data not shown).

### Liver fibrosis

Chronic liver injury typically results in the development of liver fibrosis which is characterised by extracellular deposition of collagen fibres within the liver. In support of such a process, upregulation of 3 collagen genes in FOLFOX treated animals was observed on microarray (Supplementary Table 7). Liver fibrosis is associated with transformation of quiescent hepatic stellate cells into activated myofibroblasts, whereby they express high levels of alpha smooth muscle actin (αSMA) and pro-collagen I, a process which is driven by the so called master regulator of liver fibrosis TGFβ [33]. In the liver of FOLFOX treated animals, there was upregulation of both TGFβ (3.5 fold; *p* <0.01) and procollagen I (6 fold; *p* <0.001). There was also a modest increase in αSMA expression, but this difference did not reach statistical significance (1.9 fold; *p* = 0.195; Supplementary Fig. 6A). Review of Sirius Red stained sections of the liver suggested very early collagen deposition within the injured hepatic sinusoids of FOLFOX treated animals (Supplementary Fig. 6B) although established fibrosis was not present, suggesting that this is a late event in FOLFOX induced liver injury.

## Discussion

This paper presents the first report of a reproducible experimental chronic model of oxaliplatin induced SOS, which can be used to explore in detail the pathogenesis of this condition. Prior to this report, the only model which was thought to generate similar histological features of SOS was monocrotaline administration to rats [34]. The monocrotaline model has several limitations, which the authors believe make it unsuitable for the study of oxaliplatin induced SOS, in relation to patients receiving conversion therapy for colorectal liver metastasis. The most obvious is that the stimulus driving the histological changes is different and as such there is a significant risk that the molecular mechanisms underpinning the histological changes will be distinctly different from those driving the pathology in oxaliplatin treated patients. Moreover, the development of SOS in response to monocrotaline is an acute event occurring within hours whereas oxaliplatin induced SOS occurs as a consequence of chronic drug exposure. Similarly, monocrotaline induced SOS is associated with a lobular inflammatory infiltrate, consisting of mainly neutrophils, whereas this is not described in patients treated with oxaliplatin. A further difficulty with the monocrotaline model is that its administration to rats results in the development of pulmonary hypertension which is not a feature in patients treated with oxaliplatin [35], [36]. The presence of such pathology is likely to have a significant physiological impact, thereby limiting the utility of this model in studying either the functional consequences of SOS or in the development of preventative therapeutic strategies.

An unexpected observation in the current study was that feeding a chow diet seems to protect against the development of SOS in response to FOLFOX. Chow diets are manufactured predominantly from plant materials and thereby differ considerably from purified diets, such as those used in this study, which are manufactured with refined (and defined) ingredients. A consequence of this is that chow diets may contain high concentrations of phytoestrogens (and other bioactive ingredients) which have been shown to have protective effects on the development of liver fibrosis and hepatic steatosis [37], [38]. Determining how a chow diet protects against SOS was beyond the scope of this study but further investigation of this apparent protection may help identify a potential therapeutic approach for use in patients receiving oxaliplatin based chemotherapy regimens.

PAI-1 expression was significantly elevated (x 2800 fold) in mice with FOLFOX induced SOS, suggesting it plays a central role in the pathogenesis of this condition. Indeed, PAI-1 has been implicated in all of the key pathological processes identified in this model thus far, i.e., clotting dysfunction, matrix remodelling and replicative senescence [39]. PAI-1 is transcriptionally regulated by p53, particularly in the context of senescence, which in this model occurs in response to chemotherapy induced DNA damage (within the sinusoidal endothelium). PAI-1 is not a passive marker of senescence, but rather is essential for its induction and maintenance by downregulating signalling through the mitogenic PI3K-PKB pathway utilised by many growth factors [21].

The most significant finding in the current study is that oxidative stress appears to play a major role in the pathogenesis of FOLFOX induced SOS, which can be prevented by the administration of the antioxidant BHA. The type of antioxidant used may be critical, since we have demonstrated that the thiol donor NAC is ineffective in the prevention of SOS. This is in contrast to the work by Wang *et al.* in the monocrotaline model where intraportal infusion of both glutathione and NAC was effective [28]. An explanation for this apparent disagreement may be that the route of administration chosen in our study (provision in the diet) did not allow sufficient concentrations of NAC to reach the portal circulation. However, this seems a little unlikely since NAC is absorbed readily, and rapidly transported to the liver via the portal vein [40]. The enteral route for NAC administration is more clinically relevant since the addition of daily parenteral therapy alongside systemic chemotherapy would be deemed unacceptable by patients.

In contrast, antioxidants whose effect is mediated through the activation of NRF2 appear to have the potential to prevent the development of SOS. Curcumin is one example of a clinically relevant substance which works through this pathway [41]. This is of particular interest since curcumin is currently the subject of much investigation as an adjunct to conventional chemotherapy because of its cytotoxic properties [42]. It may therefore be possible, through the targeting of NRF2, to not only reduce the incidence and severity of SOS, but also to increase the efficacy of conventional chemotherapy and this is worthy of further investigation.

We have identified the generation of a pro-thrombotic environment within the hepatic sinusoid as potentially important in the pathogenesis of SOS. Support for this hypothesis comes from the observation that patients taking regular aspirin appear to be at lower risk of developing oxaliplatin induced SOS [43]. Furthermore the fibrinolytic defibrotide has demonstrated modest success in treating patients with SOS who received myeloablative chemotherapy prior to bone marrow transplants [44]. A possible explanation for how a prothrombotic environment contributes to the development of SOS is that it results in the generation of microthrombi within the hepatic sinusoids, which subsequently become occluded, causing portal hypertension. An alternative explanation could be provided by the significant crosstalk that exists between the clotting cascade and the process of matrix remodelling. Factor Xa, acting through protease activated receptor 1 (PAR-1), can convert TGFβ from its latent to active forms [45]. It has been shown that *PAR-1* knockout mice have less fibrosis than wild types after treatment with carbon tetrachloride [46], and administration of a PAR-1 antagonist to mice subject to bile duct ligation leads to a reduction in hepatic fibrosis [47]. At present, the role of PAR-1 signalling in SOS development is uncertain, but it is conceivable that its antagonism, or indeed of Factor Xa, may lead to a reduction in perisinusoidal fibrosis seen in the severest forms of the disease and this is worthy of further exploration.

It should be noted that in the present study we have only provided hypotheses generating gene and protein expression data, which suggest a potential role for the coagulation system in the pathogenesis of SOS. To definitively establish a role for the coagulation cascade in this process, a more detailed analysis would be required, which should include focused activity assays with subsequent *in vivo* therapeutic intervention experiments, to determine the potential utility of anticoagulant strategies in preventing the development of SOS.

Two recent publications have examined gene expression changes within the liver of a cohort of patients with oxaliplatin induced SOS using microarray technology. These studies identified key processes involved in the pathogenesis of human SOS, which have also been implicated in the current model including activation of the IL-6/STAT3 pathway, activation of the coagulation system (in particular overexpression of PAI-1 and vWF), as well as overexpression of genes involved in cellular hypoxia and oxidative stress. Whilst both of these studies demonstrated upregulation of angiogenesis related genes, particularly *VEGF-C* and *VEGF-D*, we have in this experimental model demonstrated for the first time a direct association between oxaliplatin induced SOS and increased expression of VEGF-A [19], [48]. This finding adds further weight to the clinical observation that bevacizumab may be effective in reducing the incidence and severity of SOS following treatment with oxaliplatin based chemotherapy [7].

In conclusion, this paper describes a novel, reproducible model of SOS following treatment with oxaliplatin based chemotherapy, which we have used to elucidate some of the key pathophysiological processes which underpin this condition. By targeting specific pathways, it may be possible to reduce the incidence and severity of SOS without having a detrimental effect on the efficacy of systemic chemotherapy and thereby reduce the surgical morbidity after potentially curative liver resection.

## Financial support

This work was funded by a Wellcome Trust Clinical Research Training Fellowship (WT090974MA) awarded to Stuart Robinson. Steve White was funded by a Royal College of Surgeons (England) pump priming grant and a Spire Healthcare Project Grant.

Derek Mann is supported by Wellcome Trust Grants WT084961MA and WT087961MA.

## Conflict of interest

The authors who have taken part in this study declared that they do not have anything to disclose regarding funding or conflict of interest with respect to this manuscript.

## Supplementary data

Supplementary Fig. 1 **Liver injury following FOLFOX treatment was associated with an increase in serum AST and ALT (A; n = 9 per group)**. It is noteworthy that mice maintained on a chow diet do not develop histological features of SOS after 5 weeks of i.p. FOLFOX treatment (B).

Supplementary Fig. 2 **In support of cellular senescence in SOS there was an increase in PAI-1 transcript expression within the liver of FOLFOX treated animals (A; n** **=** **8 per group).** The presence of endothelial senescence was confirmed by immunohistochemistry for γH2AX (B; 20x magnification; yellow arrows) and subsequent densitometry (C; n = 9 animals per group).

Supplementary Fig. 3 **There is an increased density of PCNA staining within the liver of FOLFOX treated animals (n** **=** **9 animals per group)**

Supplementary Fig. 4 **FOLFOX induced SOS is associated with a reduction in total liver glutathione levels (A; n** **=** **6 per group).** In support of oxidative stress playing a role in the pathogenesis of SOS microarray demonstrated up-regulation of key antioxidant genes including Mt1, HO1 and SOD3 which was confirmed by qRT-PCR(B; n = 8 per group). The increase in SOD3 expression was confirmed by western blot (C; n = 6 representative animals per group). Dietary supplementation with NAC does not prevent the development of drug induced liver injury, unlike the NRF2 activating antioxidant BHA (D).

Supplementary Fig. 5 **SOS is associated with increased expression of the platelet adhesion factor vWF within the liver of FOLFOX treated mice (A; n** **=** **8 per group).** In addition review of H&E stained 2 sections of the spleen reveals the presence of large clusters of Megakaryocytes within the spleen of FOLFOX treated animals (B & C; n = 9 animals per group)suggesting that increased number of platelets may be released directly into the portal circulation. In addition further support for a pro-thrombotic in FOLFOX induced SOS is provided by the observation that Factor X transcript expression is increased within the liver of FOLFOX treated animals(D; n = 8 per group). Immunohistochemistry revealed increased expression of tissue factor, an essential component of Factor X activation, within the injured sinusoids (E; 10x magnification). Further there was increased transcript expression of the Factor X receptors PAR1 and PAR2 in the livers of these animals (F; n = 8 per group).

Supplementary Fig. 6 **FOLFOX treatment resulted in increased hepatic mRNA expression of TGFβ and procollagen 1 but not αSMA (A; n** **=** **8 per group).** Review of Sirius red stained liver sections from FOLFOX treated animals suggested early collagen deposition within the injured hepatic sinusoid (B; 20x magnification; yellow arrows) although this was not sufficient to be reflected in a significant increase in the extent of Sirius red stain detected by densitometry (C).

Supplementary Table 1 **Primer Sequences for qRT-PCR**.

Supplementary Table 2 **Antibodies for Western blot**.

Supplementary Table 3 **Antibodies for immunohistochemistry**.

Supplementary Table 4 **Histological scoring of H&E stained sections from FOLFOX treated animals and respective Vehicle Controls (n** **=** **9 per group)**.

Supplementary Table 5 **Transcripts exhibiting a 1.5 fold or greater change in expression between Control and FOLFOX treated animals**.

Supplementary Table 6 **Results of qRT-PCR to validate microarray**.

Supplementary Table 7 **Summary of genes implicated in key enriched pathways**.

Supplementary Table 8 **The effect of BHA treatment of histological scoring of liver injury**.
