# Supplementary figures and images for: Pathogenesis of FOLFOX induced sinusoidal obstruction syndrome in a murine chemotherapy model
Source: J Hepatol. 2013 Aug;59(2):318–26. doi: 10.1016/j.jhep.2013.04.014 (PMC3710969; doi:10.1016/j.jhep.2013.04.014)

**A**

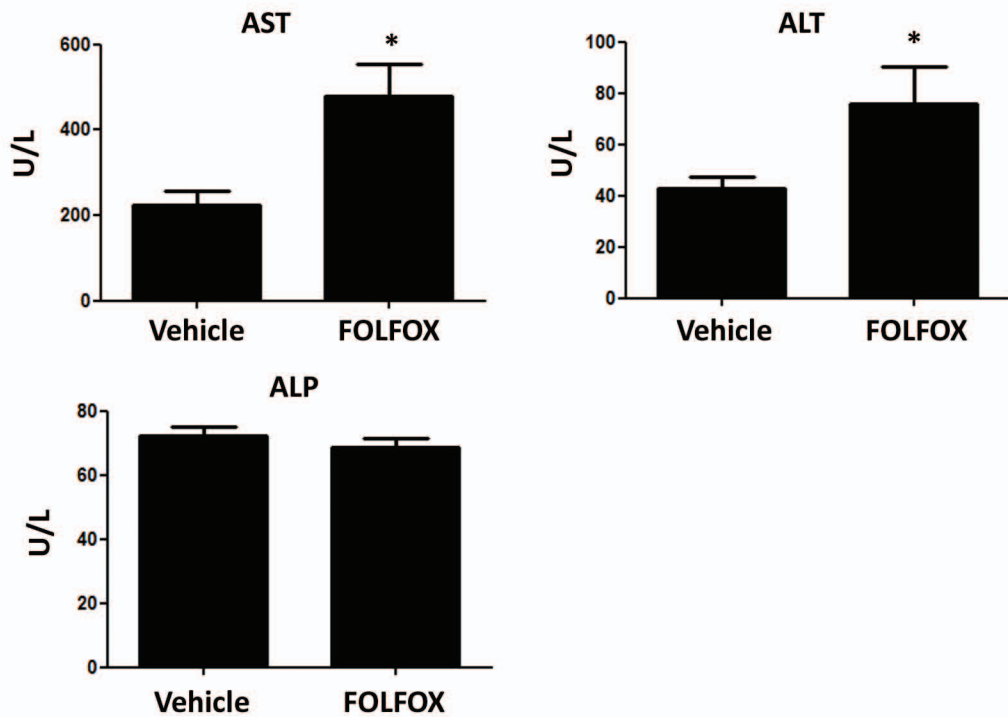

**B**

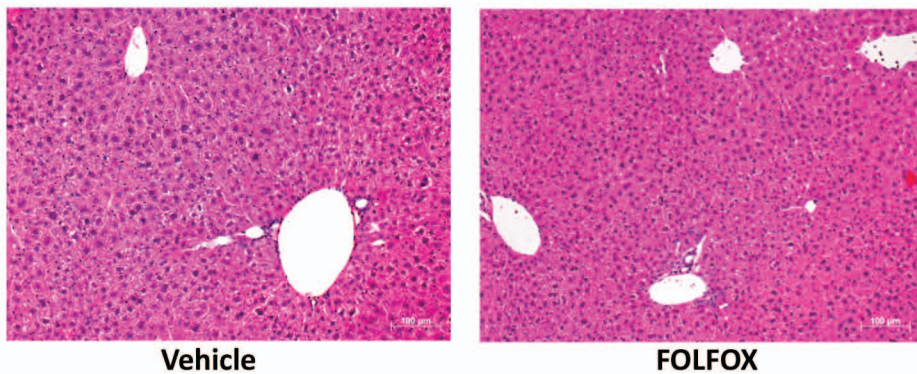

Supplement: Supplementary Fig. 1 — Liver injury following FOLFOX treatment was associated with an increase in serum AST and ALT (A; n = 9 per group). It is noteworthy that mice maintained on a chow diet do not develop histological features of SOS after 5 weeks of i.p. FOLFOX treatment (B). [file mmc1.pdf]

**A**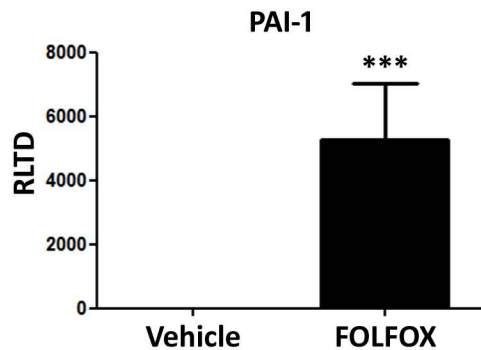**B**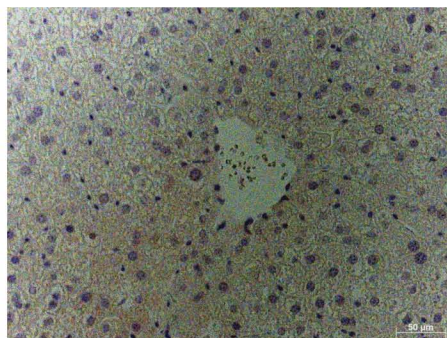**Vehicle**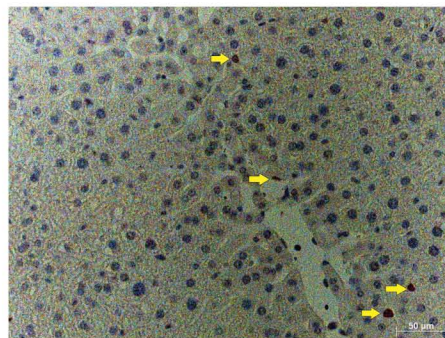**FOLFOX****C**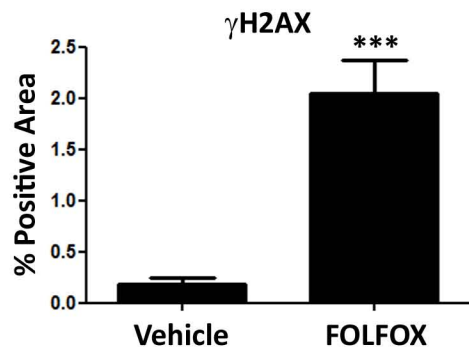

Supplement: Supplementary Fig. 2 — In support of cellular senescence in SOS there was an increase in PAI-1 transcript expression within the liver of FOLFOX treated animals (A; n = 8 per group). The presence of endothelial senescence was confirmed by immunohistochemistry for γH2AX (B; 20x magnification; yellow arrows) and subsequent densitometry (C; n = 9 animals per group). [file mmc2.pdf]

# PCNA

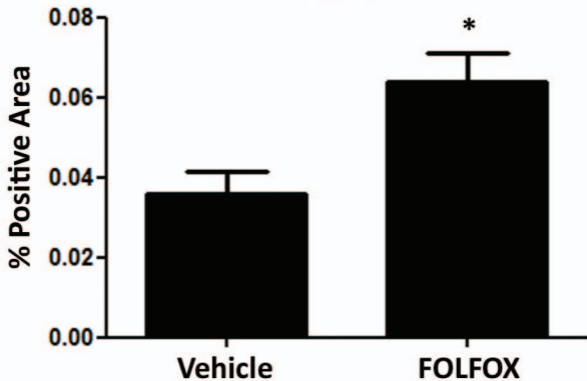

Supplement: Supplementary Fig. 3 — There is an increased density of PCNA staining within the liver of FOLFOX treated animals (n = 9 animals per group) [file mmc3.pdf]

**A**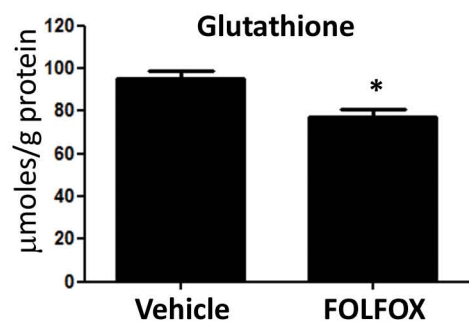**B**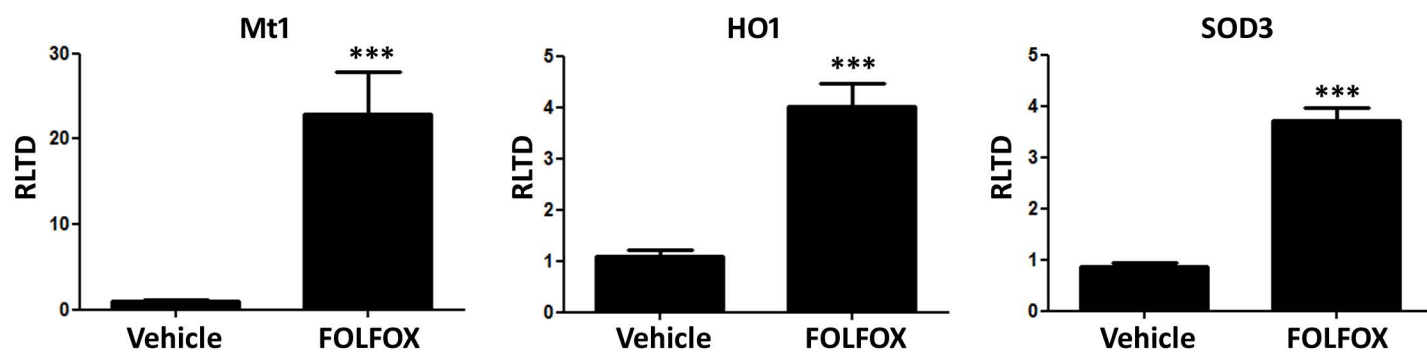**C**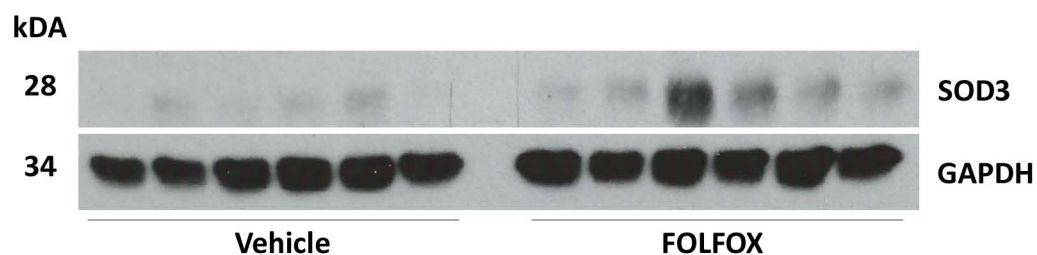**D**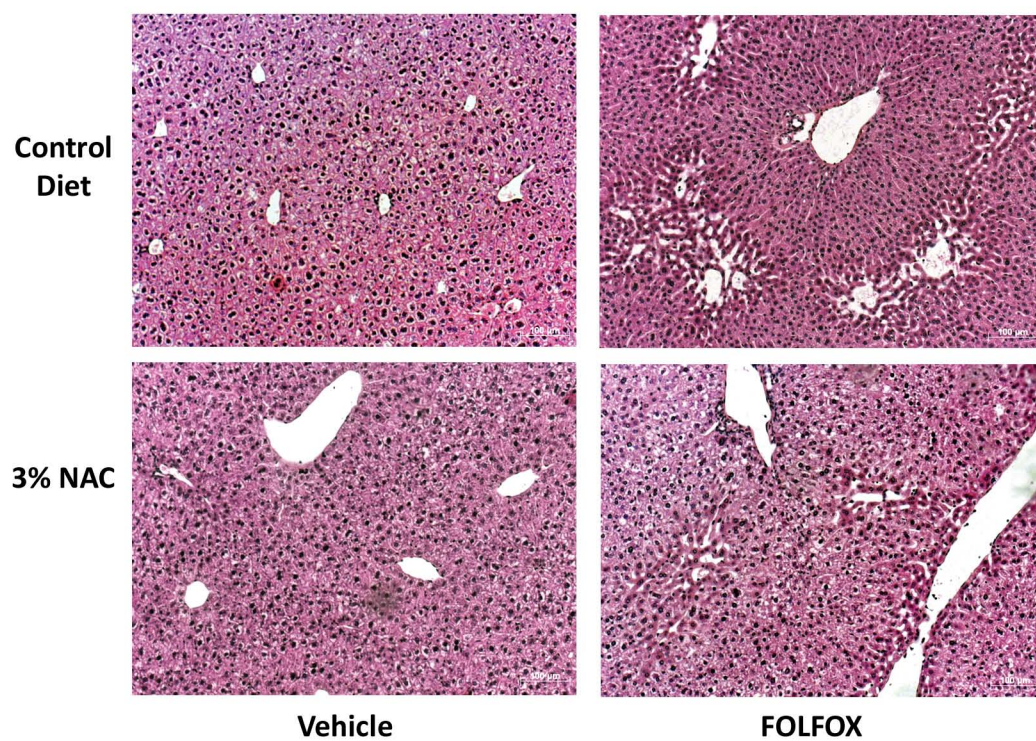

Supplement: Supplementary Fig. 4 — FOLFOX induced SOS is associated with a reduction in total liver glutathione levels (A; n = 6 per group). In support of oxidative stress playing a role in the pathogenesis of SOS microarray demonstrated up-regulation of key antioxidant genes including Mt1, HO1 and SOD3 which was confirmed by qRT-PCR(B; n = 8 per group). The increase in SOD3 expression was confirmed by western blot (C; n = 6 representative animals per group). Dietary supplementation with NAC does not prevent the development of drug induced liver injury, unlike the NRF2 activating antioxidant BHA (D). [file mmc4.pdf]

**A**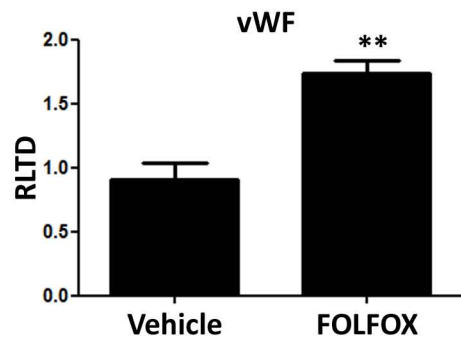**B**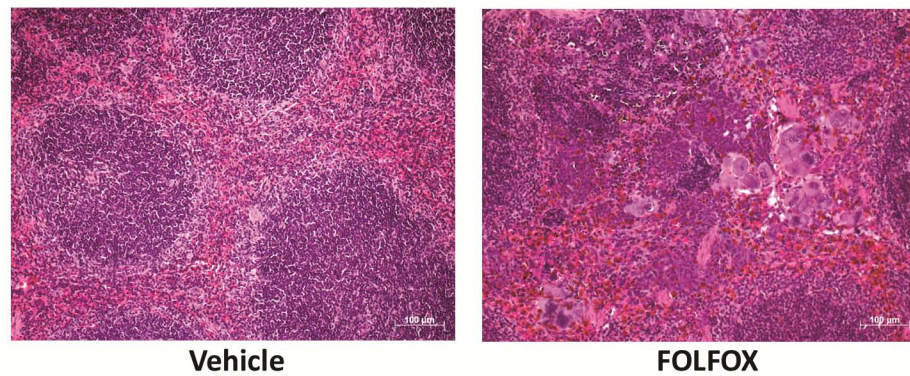**C**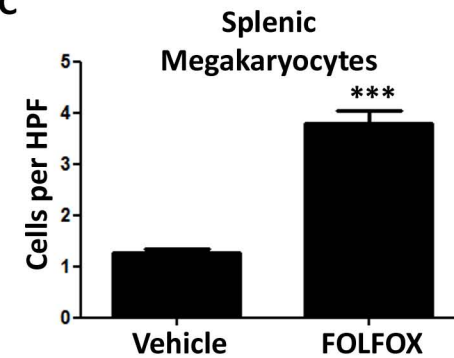**D**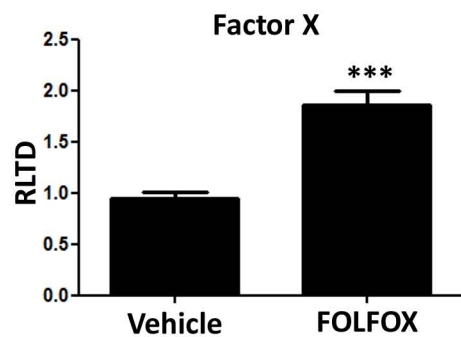**E**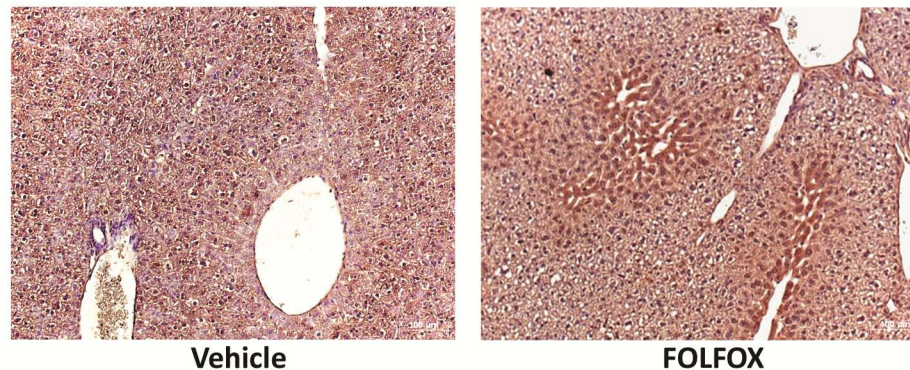**F**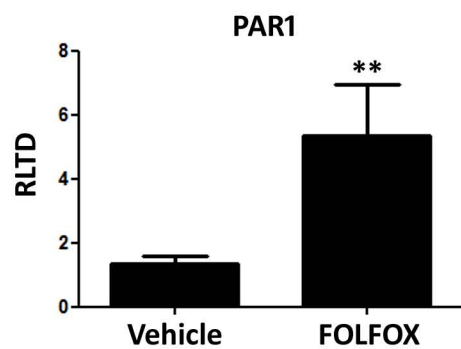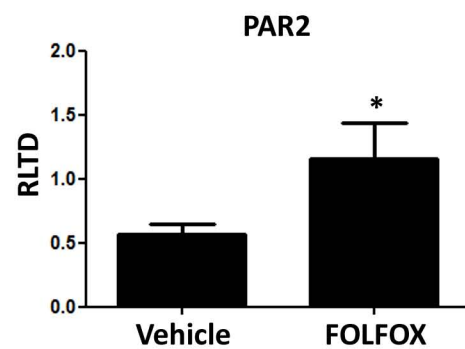

Supplement: Supplementary Fig. 5 — SOS is associated with increased expression of the platelet adhesion factor vWF within the liver of FOLFOX treated mice (A; n = 8 per group). In addition review of H&E stained 2 sections of the spleen reveals the presence of large clusters of Megakaryocytes within the spleen of FOLFOX treated animals (B & C; n = 9 animals per group)suggesting that increased number of platelets may be released directly into the portal circulation. In addition further support for a pro-thrombotic in FOLFOX induced SOS is provided by the observation that Factor X transcript expression is increased within the liver of FOLFOX treated animals(D; n = 8 per group). Immunohistochemistry revealed increased expression of tissue factor, an essential component of Factor X activation, within the injured sinusoids (E; 10x magnification). Further there was increased transcript expression of the Factor X receptors PAR1 and PAR2 in the livers of these animals (F; n = 8 per group). [file mmc5.pdf]

**A**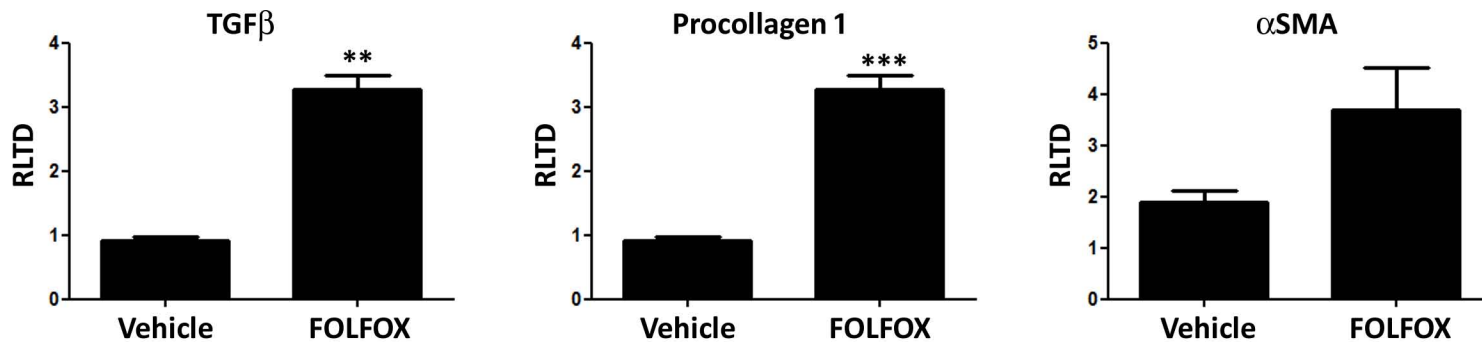**B**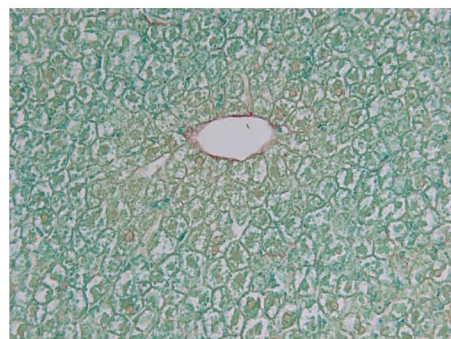**Vehicle**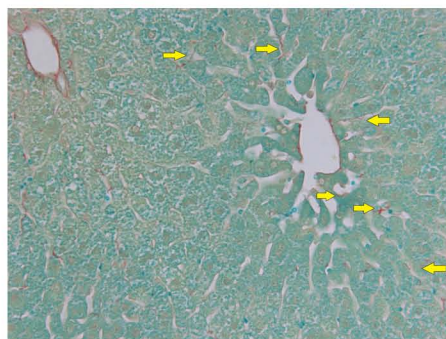**FOLFOX**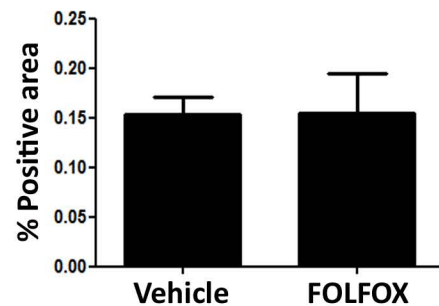

Supplement: Supplementary Fig. 6 — FOLFOX treatment resulted in increased hepatic mRNA expression of TGFβ and procollagen 1 but not αSMA (A; n = 8 per group). Review of Sirius red stained liver sections from FOLFOX treated animals suggested early collagen deposition within the injured hepatic sinusoid (B; 20x magnification; yellow arrows) although this was not sufficient to be reflected in a significant increase in the extent of Sirius red stain detected by densitometry (C). [file mmc6.pdf]
