# Supplementary material for: Pathogenesis of FOLFOX induced sinusoidal obstruction syndrome in a murine chemotherapy model
Source: J Hepatol. 2013 Aug;59(2):318–26. doi: 10.1016/j.jhep.2013.04.014 (PMC3710969; doi:10.1016/j.jhep.2013.04.014)
Supplement: Supplementary Table 1 — Primer Sequences for qRT-PCR. [file mmc7.pdf]

Product	Forward Primer Sequence	Reverse Primer Sequence
PAI-1	gatgctatgggattcaaagtca	tccacctgtttcaccatagtct
$p21^{Cip1}$	atgtccaatcctggtgatgt	tgcagcagggcagaggaagt
TXN1	catgccgaccttccagtttta	tttccttgttagcaccggaga
NQO1	tggccgaacacaagaagctg	tgggaactgaaatatcaccaggt
CXCL1	ggctgggattcacctcaag	gcgaccattcttgagtgtg
CXCL2	ccaaccaccaggctacagg	gcgtcacactcaagctctg
MCP-1	aggtccctgtcatgcttctg	tctggacccattccttcttg
IL-6	gaggataccactcccaacagacc	aagtgcatcatcgttgttcataca
Factor X	gccctaaacaccagcgacagt	gccacccacaatccgaacaag
PAR-1	ggtgctcattggctttttcta	tgccttcttcctggggtgtcc
PAR-2	ctgctgtttgtggttggtgta	cccagttgttgccattgagat
vWF	cgggaagagtgtgatggttgac	agcatctcccacagcattcacc
VEGF-A	ccccgacgagatagagta	gctttggtgaggtttgat
VEGF-B	gccagacagggttgccatac	ggagtgggatggatgatgtcag
VEGF-C	cctgggaaatgtgcctgtgaat	tgggacacagcggcatacttct
ANG1	cacatagggtgcagcaacca	cgtcgtgttctggaagaatga
ANG2	cagccacggtcaacaactc	cttctttacggatagcaaccgag
TIE2	cggccaggtacataggaggaa	tcacatctccgaacaatcagc
VEGFR-1	ccacctctctatccgctgg	accaatgtgctaaccgtcttatt
VEGFR-2	caaacctcaatgtgtctctttgc	agagtaaagcctatctcgctgt
Metallothionein 1	gctgctgctcctgctgtccc	cagcacgtgcacttgtccgc
Apolipoprotein A4	aaagtaacccagacgttcggg	cgctggatgtatggggtcag
Cyp17a1	gcccaagtcaaagacacctaat	gtacccaggcgaagagaataga
SOCS2	agttcgcattcagactacctact	tggtactcaatccgcaggttag
Cyp4a12b	ggggagatcagacccaaaagc	attcgtcggtgctgaaaccat
THRSP	atgcaagtgctaacgaaacgc	cctgccattcctcccttgg
ELOVL3	ttctcacgcgggttaaaaatgg	gagcaacagatagacgaccac
Cyp2a5	tggtcctgtattcaccatctacc	actacgccatagcctttgaaaa
ELOVL6	gaaaagcagttcaacgagaacg	agatgccgaccaccaaagata
STAT3	ctggcaccttggattgagagtc	gaaggttgtgctgatagaggac
SOD3	ccttcttgttctacggcttgc	tcgcctatcttctcaaccagg
HO1	gatagagcgcaacaagcagaa	cagtgaggcccataccagaag
MMP9	tcactttcccttcaccttcg	caaagatgaacggaacaca
TGFβ	cccgaaccccattgctgtcc	aggcgtatcagtgggggtcag
ProColl1	ttcacctacagcacgcttgtg	gatgactgtcttgccccaagtt
αSMA	tcagcgcctccagttcct	aaaaaaaaccacgagtaacaaatcaa
HES6	accacctgctagaatccatgc	gcacccggtttagttcagc
GAPDH	gcacagtcaaggccgagaat	gccttctccatggtggtgaa

Supplementary Table 1. Primer Sequences for qRT-PCR.
