# Supplementary material for: Pathogenesis of FOLFOX induced sinusoidal obstruction syndrome in a murine chemotherapy model
Source: J Hepatol. 2013 Aug;59(2):318–26. doi: 10.1016/j.jhep.2013.04.014 (PMC3710969; doi:10.1016/j.jhep.2013.04.014)
Supplement: Supplementary Table 2 — Antibodies for Western blot. [file mmc8.pdf]

Target	Concentration	Supplier	Catalogue No.
Total p53	1:1000	Abcam	31333
p-p53	1:1000	Cell Signalling Technology	9284
p21	1:1000	Abcam	7960
NRF2	1:1000	Abcam	31163
Total STAT3	1:1000	Cell Signalling Technology	9132
p-STAT3	1:1000	Cell Signalling Technology	9131
P21(Cip1)	1:1000	Abcam	7960
p-JNK	1:1000	Cell Signalling Technology	4668
Total JNK	1:1000	Cell Signalling Technology	9252
SOD3	1:500	Abcam	80946
GAPDH	1:2500	Abcam	22555

Supplementary Table 2. Antibodies for Western blot.
