# Supplementary material for: Pathogenesis of FOLFOX induced sinusoidal obstruction syndrome in a murine chemotherapy model
Source: J Hepatol. 2013 Aug;59(2):318–26. doi: 10.1016/j.jhep.2013.04.014 (PMC3710969; doi:10.1016/j.jhep.2013.04.014)
Supplement: Supplementary Table 3 — Antibodies for immunohistochemistry. [file mmc9.pdf]

Target	Antigen Retrieval	Concentration	Supplier	Catalogue No.
$p21^{Cip1}$	Citric Saline	1:100	Abcam	2961
γH2AX	EDTA pH8	1:100	Cell Signalling Technology	9718
pSTAT3	EDTA pH8	1:400	Cell Signalling Technology	9145
Tissue Factor	Citric Saline	1:100	Abcam	104513
PCNA	Trypsin	1:250	Abcam	2426

Supplementary Table 3. Antibodies for immunohistochemistry
