# Supplementary material for: Pathogenesis of FOLFOX induced sinusoidal obstruction syndrome in a murine chemotherapy model
Source: J Hepatol. 2013 Aug;59(2):318–26. doi: 10.1016/j.jhep.2013.04.014 (PMC3710969; doi:10.1016/j.jhep.2013.04.014)
Supplement: Supplementary Table 4 — Histological scoring of H&E stained sections from FOLFOX treated animals and respective Vehicle Controls (n = 9 per group). [file mmc10.pdf]

	Vehicle (n=9)	FOLFOX (n=9)	p-Value
**Rubbia-Brandt Grade**			
0	9	0	
1	0	9	p <0.0001*
2	0	0	
3	0	0	
**Endothelial Disruption Present**	0	9	p <0.0001

* = Fishers exact test comparing sinusoidal dilatation absent vs. present

Supplementary Table 4. Histological scoring of H&E stained sections from FOLFOX treated animals and respective Vehicle Controls (n=9 per group)
