# Supplementary material for: Pathogenesis of FOLFOX induced sinusoidal obstruction syndrome in a murine chemotherapy model
Source: J Hepatol. 2013 Aug;59(2):318–26. doi: 10.1016/j.jhep.2013.04.014 (PMC3710969; doi:10.1016/j.jhep.2013.04.014)
Supplement: Supplementary Table 5 — Transcripts exhibiting a 1.5 fold or greater change in expression between Control and FOLFOX treated animals. [file mmc11.pdf]

p (Corr)	p	FC (abs)	Regulation	Symbol	Entrez_Gene_ID	Definition
2.13E-04	1.08E-06	16.9	up	S100a8	20201	Mus musculus S100 calcium binding protein A8 (calgranulin A) (S100a8), mRNA.
6.17E-06	3.40E-09	12.4	up	Mt1	17748	Mus musculus metallothionein 1 (Mt1), mRNA.
4.35E-06	1.96E-09	12.0	up	Apoa4		
7.33E-05	1.51E-07	9.2	up	Lcn2	16819	Mus musculus lipocalin 2 (Lcn2), mRNA.
4.21E-07	8.44E-11	8.3	up	Apoa4	11808	Mus musculus apolipoprotein A-IV (Apoa4), mRNA.
0.001162	1.74E-05	8.3	up	S100a9	20202	Mus musculus S100 calcium binding protein A9 (calgranulin B) (S100a9), mRNA.
1.94E-05	1.73E-08	8.2	up	Cdkn1a	12575	Mus musculus cyclin-dependent kinase inhibitor 1A (P21) (Cdkn1a), mRNA.
1.09E-04	3.29E-07	7.3	up	Cyp17a1	13074	Mus musculus cytochrome P450, family 17, subfamily a, polypeptide 1 (Cyp17a1), mRNA.
1.89E-08	9.46E-13	6.9	up	Apoa4	11808	Mus musculus apolipoprotein A-IV (Apoa4), mRNA.
0.001459	2.54E-05	6.4	up	Socs2	216233	Mus musculus suppressor of cytokine signaling 2 (Socs2), mRNA.
0.031881	0.003732	6.4	up	LOC100047788	100047788	PREDICTED: Mus musculus similar to gamma-2a immunoglobulin heavy chain (LOC100047788), misc RNA.
0.001971	4.26E-05	6.3	up	Igk-C		
4.22E-05	6.56E-08	5.9	up	Serpina7	331535	Mus musculus serine (or cysteine) peptidase inhibitor, clade A (alpha-1 antiproteinase, antitrypsin), member 7 (Serpina7), mRNA.

0.003601	1.19E-04	5.5	up	LOC100047628	100047628	PREDICTED: Mus musculus similar to Chain L, Structural Basis Of Antigen Mimicry In A Clinically Relevant Melanoma Antigen System, transcript variant 3 (LOC100047628), mRNA.
0.011811	7.92E-04	5.5	up	Cyp4a14	13119	Mus musculus cytochrome P450, family 4, subfamily a, polypeptide 14 (Cyp4a14), mRNA.
9.60E-05	2.53E-07	5.3	up	Slc1a4	55963	Mus musculus solute carrier family 1 (glutamate/neutral amino acid transporter), member 4 (Slc1a4), mRNA.
4.35E-06	1.84E-09	4.3	up	Cdkn1a	12575	Mus musculus cyclin-dependent kinase inhibitor 1A (P21) (Cdkn1a), mRNA.
9.94E-04	1.38E-05	4.3	up	Gpr120	107221	Mus musculus G protein-coupled receptor 120 (Gpr120), mRNA.
3.97E-07	4.26E-11	4.2	up	Prtn3	19152	Mus musculus proteinase 3 (Prtn3), mRNA.
4.00E-05	5.62E-08	4.1	up	Fgl1	234199	Mus musculus fibrinogen-like protein 1 (Fgl1), mRNA.
3.29E-04	2.47E-06	4.1	up	Slc16a5	217316	Mus musculus solute carrier family 16 (monocarboxylic acid transporters), member 5 (Slc16a5), mRNA.
0.007526	4.02E-04	4.0	up	Gdf15	23886	Mus musculus growth differentiation factor 15 (Gdf15), mRNA.
0.005794	2.57E-04	3.9	up	Igh-VJ558	16061	PREDICTED: Mus musculus immunoglobulin heavy chain (J558 family) (Igh-VJ558), mRNA.
0.005645	2.47E-04	3.9	up	Ltf	17002	Mus musculus lactotransferrin (Ltf), mRNA.
0.017368	0.001453	3.8	up	Ighg	380794	PREDICTED: Mus musculus Immunoglobulin heavy chain (gamma polypeptide), transcript variant 1 (Ighg), mRNA.
0.042128	0.005615	3.7	up	Acot3	171281	Mus musculus acyl-CoA thioesterase 3 (Acot3), mRNA.
0.018607	0.001641	3.7	up	Egr1	13653	Mus musculus early growth response 1 (Egr1), mRNA.
4.82E-05	7.74E-08	3.6	up	Apcs	20219	Mus musculus serum amyloid P-component (Apcs), mRNA.
0.001157	1.72E-05	3.6	up	Nt5e	23959	Mus musculus 5' nucleotidase, ecto (Nt5e), mRNA.
2.89E-05	3.48E-08	3.3	up	Serpina7		
0.001	1.40E-05	3.3	up	LOC211591		
0.00368	1.25E-04	3.3	up	Cxcl1	14825	Mus musculus chemokine (C-X-C motif) ligand 1 (Cxcl1), mRNA.
0.003489	1.12E-04	3.3	up	Igfbp1	16006	Mus musculus insulin-like growth factor binding protein 1 (Igfbp1), mRNA.
2.43E-04	1.29E-06	3.3	up	Angptl3	30924	Mus musculus angiopoietin-like 3 (Angptl3), mRNA.

7.60E-07	1.90E-10	3.3	up	Pkm2	18746	Mus musculus pyruvate kinase, muscle (Pkm2), mRNA. XM_979725 XM_979753 XM_979779 XM_979805 XM_979833 XM_979863 XM_979890 XM_979922 XM_979949
5.16E-05	8.79E-08	3.1	up	Prlr	19116	Mus musculus prolactin receptor (Prlr), mRNA.
8.32E-06	5.42E-09	3.1	up	Pfkl	18641	Mus musculus phosphofructokinase, liver, B-type (Pfkl), mRNA.
1.90E-04	8.94E-07	3.1	up	Tm4sf4	229302	Mus musculus transmembrane 4 superfamily member 4 (Tm4sf4), mRNA.
0.011063	7.10E-04	3.1	up	Eraf	170812	Mus musculus erythroid associated factor (Eraf), mRNA.
0.006333	2.99E-04	3.1	up	Efna1	13636	Mus musculus ephrin A1 (Efna1), mRNA.
0.00254	6.40E-05	3.1	up	Acnat2	209186	Mus musculus acyl-coenzyme A amino acid N-acyltransferase 2 (Acnat2), mRNA.
6.26E-05	1.16E-07	3.0	up	Serpina3f	238393	Mus musculus serine (or cysteine) peptidase inhibitor, clade A, member 3F (Serpina3f), mRNA.
0.009044	5.27E-04	3.0	up	2310016C08Rik	69573	Mus musculus RIKEN cDNA 2310016C08 gene (2310016C08Rik), mRNA.
2.73E-05	2.87E-08	3.0	up	Rgs5	19737	Mus musculus regulator of G-protein signaling 5 (Rgs5), mRNA.
1.17E-05	8.82E-09	3.0	up	Fgl1		
9.37E-04	1.22E-05	3.0	up	St3gal5	20454	Mus musculus ST3 beta-galactoside alpha-2,3-sialyltransferase 5 (St3gal5), transcript variant 2, mRNA.
2.82E-05	3.11E-08	2.9	up	Rgs4	19736	Mus musculus regulator of G-protein signaling 4 (Rgs4), mRNA.
1.69E-04	7.54E-07	2.9	up	Lbp	16803	Mus musculus lipopolysaccharide binding protein (Lbp), mRNA.
4.77E-04	4.02E-06	2.8	up	Asah3l	230379	Mus musculus N-acylsphingosine amidohydrolase 3-like (Asah3l), mRNA.
0.003619	1.20E-04	2.8	up	Nlrp12	378425	PREDICTED: Mus musculus NLR family, pyrin domain containing 12 (Nlrp12), mRNA.
0.049355	0.007089	2.8	up	8430408G22Rik	213393	Mus musculus RIKEN cDNA 8430408G22 gene (8430408G22Rik), mRNA.
5.11E-04	4.51E-06	2.8	up	Serpina3g	20715	Mus musculus serine (or cysteine) peptidase inhibitor, clade A, member 3G (Serpina3g), mRNA.
1.21E-04	4.34E-07	2.7	up	Slc4a4	54403	Mus musculus solute carrier family 4 (anion exchanger), member 4 (Slc4a4), mRNA.
2.43E-04	1.30E-06	2.7	up	Egln3	112407	Mus musculus EGL nine homolog 3 (C. elegans) (Egln3), mRNA.
1.12E-04	3.63E-07	2.7	up	Il17rb	50905	Mus musculus interleukin 17 receptor B (Il17rb), mRNA.
1.83E-04	8.35E-07	2.6	up	Adam23	23792	Mus musculus a disintegrin and metallopeptidase domain 23 (Adam23),

						mRNA.
9.60E-05	2.34E-07	2.6	up	Hspa2	15512	Mus musculus heat shock protein 2 (Hspa2), transcript variant 2, mRNA.
4.38E-06	2.19E-09	2.6	up	E030030K01Rik		
0.032498	0.003832	2.6	up	Gadd45g	23882	Mus musculus growth arrest and DNA-damage-inducible 45 gamma (Gadd45g), mRNA.
0.001707	3.29E-05	2.6	up	Siat9		
9.60E-05	2.59E-07	2.6	up	Ifitm1	68713	Mus musculus interferon induced transmembrane protein 1 (Ifitm1), mRNA.
2.04E-04	9.83E-07	2.5	up	Nfkbiz	80859	Mus musculus nuclear factor of kappa light polypeptide gene enhancer in B-cells inhibitor, zeta (Nfkbiz), mRNA.
0.026214	0.00279	2.5	up	Igl-V1		
0.004135	1.53E-04	2.5	up	Cux2	13048	Mus musculus cut-like homeobox 2 (Cux2), mRNA.
0.008971	5.16E-04	2.5	up	Chac1	69065	Mus musculus ChaC, cation transport regulator-like 1 (E. coli) (Chac1), mRNA.
9.62E-04	1.30E-05	2.5	up	Lyz2	17105	Mus musculus lysozyme 2 (Lyz2), mRNA.
0.008385	4.72E-04	2.5	up	Vldlr	22359	Mus musculus very low density lipoprotein receptor (Vldlr), mRNA.
0.009969	6.07E-04	2.5	up	Ankrd37	654824	Mus musculus ankyrin repeat domain 37 (Ankrd37), mRNA.
3.97E-07	5.97E-11	2.4	up	Phlda3	27280	Mus musculus pleckstrin homology-like domain, family A, member 3 (Phlda3), mRNA.
7.79E-05	1.64E-07	2.4	up	4833418A01Rik	75763	Mus musculus RIKEN cDNA 4833418A01 gene (4833418A01Rik), mRNA.
2.50E-04	1.42E-06	2.4	up	Hmox1	15368	Mus musculus heme oxygenase (decycling) 1 (Hmox1), mRNA.
0.002461	6.07E-05	2.4	up	LOC100043257	100043257	PREDICTED: Mus musculus similar to RNA binding motif protein 3 (LOC100043257), mRNA.
3.93E-05	5.32E-08	2.3	up	Cyp3a13	13113	Mus musculus cytochrome P450, family 3, subfamily a, polypeptide 13 (Cyp3a13), mRNA.
1.72E-04	7.75E-07	2.3	up	Bsg		
3.19E-04	2.21E-06	2.3	up	Akr1b8	14187	Mus musculus aldo-keto reductase family 1, member B8 (Akr1b8), mRNA.
2.10E-04	1.04E-06	2.3	up	Cyp3a13	13113	Mus musculus cytochrome P450, family 3, subfamily a, polypeptide 13 (Cyp3a13), mRNA.

9.60E-05	2.59E-07	2.3	up	Serpina3m	20717	Mus musculus serine (or cysteine) peptidase inhibitor, clade A, member 3M (Serpina3m), mRNA.
0.01916	0.001707	2.3	up	Slc4a1	20533	Mus musculus solute carrier family 4 (anion exchanger), member 1 (Slc4a1), mRNA.
1.25E-04	4.90E-07	2.3	up	Stab2	192188	Mus musculus stabilin 2 (Stab2), mRNA.
4.08E-04	3.17E-06	2.3	up	Ccdc80	67896	Mus musculus coiled-coil domain containing 80 (Ccdc80), mRNA.
0.002756	7.32E-05	2.3	up	D1Ertd471e		
0.002967	8.28E-05	2.3	up	Klf6	23849	Mus musculus Kruppel-like factor 6 (Klf6), mRNA.
6.67E-04	7.42E-06	2.3	up	Lyve1	114332	Mus musculus lymphatic vessel endothelial hyaluronan receptor 1 (Lyve1), mRNA.
0.011512	7.58E-04	2.2	up	Npal1	70701	Mus musculus NIPA-like domain containing 1 (Npal1), mRNA.
0.001611	3.02E-05	2.2	up	Marco		
0.013467	9.89E-04	2.2	up	Bhmt	12116	Mus musculus betaine-homocysteine methyltransferase (Bhmt), mRNA.
0.013379	9.75E-04	2.2	up	Irs2	384783	Mus musculus insulin receptor substrate 2 (Irs2), mRNA.
2.34E-06	8.21E-10	2.2	up	Tnfrsf21	94185	Mus musculus tumor necrosis factor receptor superfamily, member 21 (Tnfrsf21), mRNA.
0.006154	2.84E-04	2.2	up	Cbr1	12408	Mus musculus carbonyl reductase 1 (Cbr1), mRNA.
1.12E-04	3.75E-07	2.2	up	Serpina3n	20716	Mus musculus serine (or cysteine) peptidase inhibitor, clade A, member 3N (Serpina3n), mRNA.
0.003435	1.08E-04	2.2	up	Rapgef4	56508	Mus musculus Rap guanine nucleotide exchange factor (GEF) 4 (Rapgef4), mRNA.
8.97E-04	1.15E-05	2.2	up	Dnajc12	30045	Mus musculus DnaJ (Hsp40) homolog, subfamily C, member 12 (Dnajc12), mRNA.
5.06E-05	8.37E-08	2.2	up	Lyz	17110	Mus musculus lysozyme (Lyz), mRNA.
0.001307	2.14E-05	2.2	up	Ela2a	13706	Mus musculus elastase 2A (Ela2a), mRNA.
0.049716	0.007169	2.2	up	Ddit4	74747	Mus musculus DNA-damage-inducible transcript 4 (Ddit4), mRNA.
0.029683	0.003344	2.2	up	Igk-V5	381777	PREDICTED: Mus musculus immunoglobulin kappa chain variable 5 (V5 family) (Igk-V5), mRNA.
0.004256	1.60E-04	2.1	up	Tmem176a	66058	Mus musculus transmembrane protein 176A (Tmem176a), mRNA.
0.025735	0.002714	2.1	up	Camp	12796	Mus musculus cathelicidin antimicrobial peptide (Camp), mRNA.

0.003215	9.60E-05	2.1	up	Retnlg	245195	Mus musculus resistin like gamma (Retnlg), mRNA.
0.003601	1.19E-04	2.1	up	Tgm2	21817	Mus musculus transglutaminase 2, C polypeptide (Tgm2), mRNA.
0.012886	9.18E-04	2.1	up	2200001I15Rik	69134	Mus musculus RIKEN cDNA 2200001I15 gene (2200001I15Rik), mRNA.
0.015292	0.001206	2.1	up	Cd63		
1.58E-04	6.60E-07	2.1	up	Esam	69524	Mus musculus endothelial cell-specific adhesion molecule (Esam), mRNA.
1.25E-04	4.58E-07	2.1	up	Ier3	15937	Mus musculus immediate early response 3 (Ier3), mRNA.
0.005162	2.14E-04	2.1	up	LOC100046232	100046232	PREDICTED: Mus musculus similar to NFIL3/E4BP4 transcription factor (LOC100046232), mRNA.
0.004532	1.75E-04	2.1	up	Onecut2	225631	Mus musculus one cut domain, family member 2 (Onecut2), mRNA.
0.001608	3.00E-05	2.1	up	Lgals3	16854	Mus musculus lectin, galactose binding, soluble 3 (Lgals3), mRNA.
0.037156	0.004655	2.1	up	LOC381774		
0.001385	2.35E-05	2.1	up	Aldoc	11676	Mus musculus aldolase C, fructose-bisphosphate (Aldoc), mRNA.
3.27E-04	2.35E-06	2.1	up	Mbl2	17195	Mus musculus mannose-binding lectin (protein C) 2 (Mbl2), mRNA.
0.005779	2.55E-04	2.1	up	Por	18984	Mus musculus P450 (cytochrome) oxidoreductase (Por), mRNA.
0.001645	3.11E-05	2.1	up	Sgk1	20393	Mus musculus serum/glucocorticoid regulated kinase 1 (Sgk1), mRNA.
0.01858	0.001636	2.1	up	Vldlr	22359	Mus musculus very low density lipoprotein receptor (Vldlr), mRNA.
7.70E-04	9.15E-06	2.1	up	Gja1	14609	Mus musculus gap junction membrane channel protein alpha 1 (Gja1), mRNA.
1.12E-04	3.68E-07	2.0	up	Abcc9	20928	Mus musculus ATP-binding cassette, sub-family C (CFTR/MRP), member 9 (Abcc9), transcript variant 4, mRNA.
0.007421	3.93E-04	2.0	up	Nt5e	23959	Mus musculus 5' nucleotidase, ecto (Nt5e), mRNA.
0.001432	2.45E-05	2.0	up	Fkbp5	14229	Mus musculus FK506 binding protein 5 (Fkbp5), mRNA.
0.009193	5.40E-04	2.0	up	Ngp	18054	Mus musculus neutrophilic granule protein (Ngp), mRNA.
0.019817	0.001802	2.0	up	Id1	15901	Mus musculus inhibitor of DNA binding 1 (Id1), mRNA.
6.58E-05	1.25E-07	2.0	up	Dsg1c		
0.028905	0.003233	2.0	up	Vldlr	22359	Mus musculus very low density lipoprotein receptor (Vldlr), mRNA.
0.005544	2.39E-04	2.0	up	Por	18984	Mus musculus P450 (cytochrome) oxidoreductase (Por), mRNA.
0.001471	2.61E-05	2.0	up	Pik3ap1	83490	Mus musculus phosphoinositide-3-kinase adaptor protein 1 (Pik3ap1), mRNA.

3.29E-04	2.49E-06	2.0	up	Gpx3	14778	Mus musculus glutathione peroxidase 3 (Gpx3), transcript variant 2, mRNA.
7.20E-04	8.25E-06	2.0	up	OTTMUSG00000000971	100034251	Mus musculus predicted gene, OTTMUSG00000000971 (OTTMUSG00000000971), mRNA.
1.25E-04	4.69E-07	2.0	up	Fgf1	14164	Mus musculus fibroblast growth factor 1 (Fgf1), mRNA.
0.042266	0.005645	2.0	up	Txnip	56338	Mus musculus thioredoxin interacting protein (Txnip), transcript variant 2, mRNA.
1.25E-04	4.68E-07	2.0	up	Prhoxnb	231903	Mus musculus parahox cluster neighbor (Prhoxnb), mRNA.
2.54E-04	1.49E-06	2.0	up	Mmp14	17387	Mus musculus matrix metallopeptidase 14 (membrane-inserted) (Mmp14), mRNA.
0.001384	2.34E-05	2.0	up	BC048546	232400	Mus musculus cDNA sequence BC048546 (BC048546), mRNA.
8.32E-05	1.89E-07	2.0	up	Slc40a1	53945	Mus musculus solute carrier family 40 (iron-regulated transporter), member 1 (Slc40a1), mRNA.
0.001268	2.05E-05	2.0	up	Trp53inp1	60599	Mus musculus transformation related protein 53 inducible nuclear protein 1 (Trp53inp1), mRNA.
3.80E-04	2.92E-06	2.0	up	Cdkn1a	12575	Mus musculus cyclin-dependent kinase inhibitor 1A (P21) (Cdkn1a), mRNA.
6.29E-04	6.41E-06	2.0	up	Bsg	12215	Mus musculus basigin (Bsg), transcript variant 1, mRNA.
0.003352	1.03E-04	2.0	up	Nudt18	213484	Mus musculus nudix (nucleoside diphosphate linked moiety X)-type motif 18 (Nudt18), mRNA.
0.010744	6.80E-04	2.0	up	Igh-VJ558		
7.12E-05	1.39E-07	1.9	up	Cxcl4	56744	Mus musculus chemokine (C-X-C motif) ligand 4 (Cxcl4), mRNA.
1.27E-04	5.11E-07	1.9	up	Kcnj8	16523	Mus musculus potassium inwardly-rectifying channel, subfamily J, member 8 (Kcnj8), mRNA.
6.32E-04	6.78E-06	1.9	up	Nrp1	18186	Mus musculus neuropilin 1 (Nrp1), mRNA.
2.44E-04	1.35E-06	1.9	up	Cd93	17064	Mus musculus CD93 antigen (Cd93), mRNA.
5.22E-04	4.95E-06	1.9	up	BC048546	232400	Mus musculus cDNA sequence BC048546 (BC048546), mRNA.
0.002206	5.13E-05	1.9	up	Col6a1	12833	Mus musculus procollagen, type VI, alpha 1 (Col6a1), mRNA.
0.00647	3.09E-04	1.9	up	1200016E24Rik		
8.90E-06	6.25E-09	1.9	up	Msc	17681	Mus musculus musculin (Msc), mRNA.
1.20E-04	4.14E-07	1.9	up	Prhoxnb	231903	Mus musculus parahox cluster neighbor (Prhoxnb), mRNA.

0.018621	0.001645	1.9	up	Peci	23986	Mus musculus peroxisomal delta3, delta2-enoyl-Coenzyme A isomerase (Peci), mRNA.
9.73E-04	1.33E-05	1.9	up	Cyp3a13	13113	Mus musculus cytochrome P450, family 3, subfamily a, polypeptide 13 (Cyp3a13), mRNA.
2.89E-05	3.47E-08	1.9	up	Itih3	16426	Mus musculus inter-alpha trypsin inhibitor, heavy chain 3 (Itih3), mRNA.
0.001145	1.69E-05	1.9	up	Dnajc12	30045	Mus musculus DnaJ (Hsp40) homolog, subfamily C, member 12 (Dnajc12), mRNA.
0.032973	0.003915	1.9	up	Slc38a2	67760	Mus musculus solute carrier family 38, member 2 (Slc38a2), mRNA.
0.003884	1.38E-04	1.9	up	LOC100044948	100044948	PREDICTED: Mus musculus similar to macrophage migration inhibitory factor (LOC100044948), mRNA.
0.001582	2.89E-05	1.9	up	Rhob	11852	Mus musculus ras homolog gene family, member B (Rhob), mRNA.
0.013467	9.89E-04	1.9	up	9030619P08Rik	105892	Mus musculus RIKEN cDNA 9030619P08 gene (9030619P08Rik), mRNA.
0.002206	5.14E-05	1.9	up	Rnf186	66825	Mus musculus ring finger protein 186 (Rnf186), mRNA.
0.012287	8.46E-04	1.9	up	C730009D12		
0.003527	1.15E-04	1.9	up	Cyp2c54	404195	Mus musculus cytochrome P450, family 2, subfamily c, polypeptide 54 (Cyp2c54), mRNA.
0.002174	5.00E-05	1.8	up	Nrp1	18186	Mus musculus neuropilin 1 (Nrp1), mRNA.
0.012146	8.25E-04	1.8	up	S100a6	20200	Mus musculus S100 calcium binding protein A6 (calcyclin) (S100a6), mRNA.
7.28E-05	1.46E-07	1.8	up	Hp	15439	Mus musculus haptoglobin (Hp), mRNA.
3.15E-04	2.16E-06	1.8	up	Stat3	20848	Mus musculus signal transducer and activator of transcription 3 (Stat3), transcript variant 1, mRNA.
0.012128	8.23E-04	1.8	up	Lrfn3	233067	Mus musculus leucine rich repeat and fibronectin type III domain containing 3 (Lrfn3), mRNA.
0.003311	1.01E-04	1.8	up	Ugcg	22234	Mus musculus UDP-glucose ceramide glucosyltransferase (Ugcg), mRNA.
9.60E-05	2.56E-07	1.8	up	B230365C01Rik		
5.82E-04	5.77E-06	1.8	up	Apoa5	66113	Mus musculus apolipoprotein A-V (Apoa5), mRNA.
4.40E-04	3.55E-06	1.8	up	LOC100044204	100044204	PREDICTED: Mus musculus hypothetical protein LOC100044204 (LOC100044204), mRNA.
0.011512	7.58E-04	1.8	up	2310040A07Rik	69638	PREDICTED: Mus musculus RIKEN cDNA 2310040A07 gene (2310040A07Rik), mRNA.

0.006813	3.41E-04	1.8	up	Slco1a4	28250	Mus musculus solute carrier organic anion transporter family, member 1a4 (Slco1a4), mRNA.
4.22E-05	6.40E-08	1.8	up	C920030L09Rik		
6.29E-04	6.57E-06	1.8	up	2810410P22Rik		
0.005972	2.72E-04	1.8	up	Ugcg		
1.88E-04	8.66E-07	1.8	up	Phlda3	27280	Mus musculus pleckstrin homology-like domain, family A, member 3 (Phlda3), mRNA.
0.01789	0.001529	1.8	up	Cyp3a11	13112	Mus musculus cytochrome P450, family 3, subfamily a, polypeptide 11 (Cyp3a11), mRNA.
9.60E-05	2.58E-07	1.8	up	Hp	15439	Mus musculus haptoglobin (Hp), mRNA.
0.025201	0.002627	1.8	up	Pex11a	18631	Mus musculus peroxisomal biogenesis factor 11a (Pex11a), mRNA.
5.11E-04	4.54E-06	1.8	up	Ldha	16828	Mus musculus lactate dehydrogenase A (Ldha), mRNA.
0.009088	5.32E-04	1.8	up	LOC634731	634731	PREDICTED: Mus musculus similar to sushi domain containing 1 (LOC634731), mRNA.
0.001222	1.93E-05	1.8	up	Avpr1a	54140	Mus musculus arginine vasopressin receptor 1A (Avpr1a), mRNA.
0.001365	2.27E-05	1.8	up	Tnfrsf11b	18383	Mus musculus tumor necrosis factor receptor superfamily, member 11b (osteoprotegerin) (Tnfrsf11b), mRNA.
0.025803	0.002732	1.8	up	Pex11a	18631	Mus musculus peroxisomal biogenesis factor 11a (Pex11a), mRNA.
0.001469	2.59E-05	1.8	up	Colec11	71693	Mus musculus collectin sub-family member 11 (Colec11), mRNA.
8.98E-04	1.16E-05	1.8	up	Stat3	20848	Mus musculus signal transducer and activator of transcription 3 (Stat3), transcript variant 1, mRNA.
0.008697	4.96E-04	1.8	up	4933407C03Rik	74440	PREDICTED: Mus musculus RIKEN cDNA 4933407C03 gene, transcript variant 6 (4933407C03Rik), mRNA.
0.006689	3.29E-04	1.8	up	Mfsd7c	217721	Mus musculus major facilitator superfamily domain containing 7C (Mfsd7c), mRNA.
0.002835	7.70E-05	1.8	up	Col4a1	12826	Mus musculus procollagen, type IV, alpha 1 (Col4a1), mRNA.
0.003636	1.22E-04	1.7	up	AW112037		
0.043972	0.005986	1.7	up	Anxa2	12306	Mus musculus annexin A2 (Anxa2), mRNA.
0.011841	7.95E-04	1.7	up	1810011O10Rik	69068	Mus musculus RIKEN cDNA 1810011O10 gene (1810011O10Rik), mRNA.
4.95E-04	4.32E-06	1.7	up	2810410P22Rik		

6.37E-04	6.89E-06	1.7	up	Arl5a	75423	Mus musculus ADP-ribosylation factor-like 5A (Arl5a), mRNA.
0.017255	0.001438	1.7	up	Cited2	17684	Mus musculus Cbp/p300-interacting transactivator, with Glu/Asp-rich carboxy-terminal domain, 2 (Cited2), mRNA.
0.004473	1.70E-04	1.7	up	1810058I24Rik	67705	PREDICTED: Mus musculus RIKEN cDNA 1810058I24 gene (1810058I24Rik), misc RNA.
8.22E-05	1.77E-07	1.7	up	Zmat3	22401	Mus musculus zinc finger matrin type 3 (Zmat3), mRNA.
0.030394	0.003449	1.7	up	Gas6	14456	Mus musculus growth arrest specific 6 (Gas6), mRNA.
0.002518	6.30E-05	1.7	up	Egfl7	353156	Mus musculus EGF-like domain 7 (Egfl7), transcript variant c, mRNA.
9.58E-04	1.28E-05	1.7	up	Mmrn2	105450	Mus musculus multimerin 2 (Mmrn2), mRNA.
0.01131	7.37E-04	1.7	up	Icam1	15894	Mus musculus intercellular adhesion molecule 1 (Icam1), mRNA.
2.43E-04	1.32E-06	1.7	up	Zfp36l1	12192	Mus musculus zinc finger protein 36, C3H type-like 1 (Zfp36l1), mRNA.
9.69E-04	1.32E-05	1.7	up	2310061J03Rik		
0.026409	0.00283	1.7	up	Slc17a8	216227	Mus musculus solute carrier family 17 (sodium-dependent inorganic phosphate cotransporter), member 8 (Slc17a8), mRNA.
6.29E-04	6.47E-06	1.7	up	Arrdc4	66412	Mus musculus arrestin domain containing 4 (Arrdc4), transcript variant 1, mRNA.
0.015393	0.001219	1.7	up	Cd63	12512	Mus musculus CD63 antigen (Cd63), transcript variant 2, mRNA.
0.003065	8.81E-05	1.7	up	Slc11a2		
0.025051	0.002604	1.7	up	Mgst3	66447	Mus musculus microsomal glutathione S-transferase 3 (Mgst3), mRNA.
0.004951	2.01E-04	1.7	up	Vcam1	22329	Mus musculus vascular cell adhesion molecule 1 (Vcam1), mRNA.
0.032471	0.003826	1.7	up	Gadd45b	17873	Mus musculus growth arrest and DNA-damage-inducible 45 beta (Gadd45b), mRNA.
0.045882	0.00638	1.7	up	Pex11a		
3.93E-05	5.09E-08	1.7	up	Gulo	268756	Mus musculus gulonolactone (L-) oxidase (Gulo), mRNA.
9.64E-05	2.66E-07	1.7	up	Lrg1	76905	Mus musculus leucine-rich alpha-2-glycoprotein 1 (Lrg1), mRNA.
4.29E-04	3.42E-06	1.7	up	Tmem176b	65963	Mus musculus transmembrane protein 176B (Tmem176b), mRNA.
0.00739	3.90E-04	1.7	up	4933407C03Rik	74440	PREDICTED: Mus musculus RIKEN cDNA 4933407C03 gene, transcript variant 5 (4933407C03Rik), mRNA.
0.003102	9.09E-05	1.7	up	Sparc	20692	Mus musculus secreted acidic cysteine rich glycoprotein (Sparc), mRNA.

0.00115	1.71E-05	1.7	up	Ms4a6d	68774	Mus musculus membrane-spanning 4-domains, subfamily A, member 6D (Ms4a6d), mRNA.
0.002147	4.83E-05	1.7	up	Ppap2c	50784	Mus musculus phosphatidic acid phosphatase type 2c (Ppap2c), mRNA.
0.004766	1.88E-04	1.7	up	Nfkbia	18035	Mus musculus nuclear factor of kappa light polypeptide gene enhancer in B-cells inhibitor, alpha (Nfkbia), mRNA.
5.61E-04	5.45E-06	1.7	up	Ednra	13617	Mus musculus endothelin receptor type A (Ednra), mRNA.
0.010281	6.35E-04	1.7	up	Clpx		
0.00684	3.46E-04	1.7	up	Fas	14102	Mus musculus Fas (TNF receptor superfamily member 6) (Fas), mRNA.
0.048296	0.006895	1.7	up	S100a11	20195	Mus musculus S100 calcium binding protein A11 (calgizzarin) (S100a11), mRNA.
1.07E-04	3.09E-07	1.7	up	Cyba	13057	Mus musculus cytochrome b-245, alpha polypeptide (Cyba), mRNA.
0.017639	0.001493	1.7	up	Paqr9	75552	Mus musculus progestin and adipoQ receptor family member IX (Paqr9), mRNA.
0.004015	1.46E-04	1.7	up	Tmem86a	67893	Mus musculus transmembrane protein 86A (Tmem86a), mRNA.
0.011121	7.16E-04	1.6	up	Lyzs	17105	Mus musculus lysozyme (Lyzs), mRNA.
6.29E-04	6.68E-06	1.6	up	Nrn1	68404	Mus musculus neuritin 1 (Nrn1), mRNA.
9.58E-04	1.28E-05	1.6	up	Dab2		
1.71E-06	5.13E-10	1.6	up	Pcdh17	219228	Mus musculus protocadherin 17 (Pcdh17), mRNA.
0.003311	1.00E-04	1.6	up	1500012F01Rik	68949	Mus musculus RIKEN cDNA 1500012F01 gene (1500012F01Rik), mRNA.
0.00646	3.08E-04	1.6	up	Cygb	114886	Mus musculus cytoglobin (Cygb), mRNA.
1.60E-04	7.06E-07	1.6	up	E130203B14Rik	320736	Mus musculus RIKEN cDNA E130203B14 gene (E130203B14Rik), mRNA.
0.008207	4.57E-04	1.6	up	Pbld	68371	Mus musculus phenazine biosynthesis-like protein domain containing (Pbld), mRNA.
0.005413	2.29E-04	1.6	up	Ptp4a1	19243	Mus musculus protein tyrosine phosphatase 4a1 (Ptp4a1), mRNA.
0.036612	0.004549	1.6	up	2310076L09Rik	66968	Mus musculus RIKEN cDNA 2310076L09 gene (2310076L09Rik), transcript variant 1, mRNA.
1.89E-04	8.81E-07	1.6	up	Dab2	13132	Mus musculus disabled homolog 2 (Drosophila) (Dab2), transcript variant 2, mRNA.
0.005425	2.31E-04	1.6	up	Ear3	53876	Mus musculus eosinophil-associated, ribonuclease A family, member 3 (Ear3), mRNA.

0.003374	1.05E-04	1.6	up	Fn1	14268	Mus musculus fibronectin 1 (Fn1), mRNA.
0.040559	0.005302	1.6	up	Sfrs5	20384	Mus musculus splicing factor, arginine/serine-rich 5 (SRp40, HRS) (Sfrs5), transcript variant 2, mRNA.
0.001278	2.09E-05	1.6	up	Tgfbi	21810	Mus musculus transforming growth factor, beta induced (Tgfbi), mRNA.
0.00404	1.48E-04	1.6	up	Sparc	20692	Mus musculus secreted acidic cysteine rich glycoprotein (Sparc), mRNA.
9.47E-04	1.25E-05	1.6	up	Eps8l2	98845	Mus musculus EPS8-like 2 (Eps8l2), mRNA.
0.001147	1.70E-05	1.6	up	Fetub	59083	Mus musculus fetuin beta (Fetub), transcript variant 1, mRNA.
1.58E-04	6.75E-07	1.6	up	Bnip3l	12177	Mus musculus BCL2/adenovirus E1B interacting protein 3-like (Bnip3l), mRNA.
0.012249	8.38E-04	1.6	up	Litaf	56722	Mus musculus LPS-induced TN factor (Litaf), mRNA.
0.001931	4.10E-05	1.6	up	Abca8b	27404	Mus musculus ATP-binding cassette, sub-family A (ABC1), member 8b (Abca8b), mRNA.
0.001885	3.87E-05	1.6	up	Pcp4l1	66425	PREDICTED: Mus musculus Purkinje cell protein 4-like 1 (Pcp4l1), mRNA.
0.013025	9.36E-04	1.6	up	Lgmn	19141	Mus musculus legumain (Lgmn), mRNA.
0.003901	1.40E-04	1.6	up	S3-12	57435	Mus musculus plasma membrane associated protein, S3-12 (S3-12), mRNA.
0.026358	0.002816	1.6	up	EG240549	240549	Mus musculus predicted gene, EG240549 (EG240549), mRNA.
0.037676	0.004762	1.6	up	Paqr9	75552	Mus musculus progestin and adipoQ receptor family member IX (Paqr9), mRNA.
0.011334	7.39E-04	1.6	up	Ung	22256	Mus musculus uracil DNA glycosylase (Ung), transcript variant 2, mRNA.
0.002704	7.11E-05	1.6	up	Abhd2	54608	Mus musculus abhydrolase domain containing 2 (Abhd2), mRNA.
4.29E-04	3.44E-06	1.6	up	Stat3	20848	Mus musculus signal transducer and activator of transcription 3 (Stat3), transcript variant 3, mRNA.
9.60E-04	1.29E-05	1.6	up	Csrp3	13009	Mus musculus cysteine and glycine-rich protein 3 (Csrp3), mRNA.
2.94E-04	1.90E-06	1.6	up	BC025446	223631	Mus musculus cDNA sequence BC025446 (BC025446), mRNA.
0.00885	5.07E-04	1.6	up	Parp16	214424	Mus musculus poly (ADP-ribose) polymerase family, member 16 (Parp16), mRNA.
0.015908	0.001276	1.6	up	B3galt1	26877	Mus musculus UDP-Gal:betaGlcNAc beta 1,3-galactosyltransferase, polypeptide 1 (B3galt1), mRNA.
0.003901	1.40E-04	1.6	up	Flt1	14254	Mus musculus FMS-like tyrosine kinase 1 (Flt1), mRNA.
0.003311	1.01E-04	1.6	up	Robo1	19876	Mus musculus roundabout homolog 1 (Drosophila) (Robo1), mRNA.

0.008636	4.90E-04	1.6	up	Pla2g12a	66350	Mus musculus phospholipase A2, group XIIA (Pla2g12a), transcript variant 1, mRNA.
0.005164	2.15E-04	1.6	up	Ppic	19038	Mus musculus peptidylprolyl isomerase C (Ppic), mRNA.
2.68E-04	1.63E-06	1.6	up	Kng2	385643	Mus musculus kininogen 2 (Kng2), mRNA.
4.95E-04	4.30E-06	1.6	up	Sod3	20657	Mus musculus superoxide dismutase 3, extracellular (Sod3), mRNA.
0.009929	6.02E-04	1.6	up	Nr1i3	12355	Mus musculus nuclear receptor subfamily 1, group I, member 3 (Nr1i3), mRNA.
0.026414	0.002832	1.6	up	2310040A07Rik	69638	PREDICTED: Mus musculus RIKEN cDNA 2310040A07 gene (2310040A07Rik), mRNA.
0.00616	2.85E-04	1.6	up	Abca8		
0.004473	1.71E-04	1.6	up	Prkcbp1	228880	Mus musculus protein kinase C binding protein 1 (Prkcbp1), mRNA.
0.001206	1.87E-05	1.6	up	Apobec1	11810	Mus musculus apolipoprotein B mRNA editing enzyme, catalytic polypeptide 1 (Apobec1), transcript variant 1, mRNA.
0.006527	3.17E-04	1.6	up	Trp53inp1	60599	Mus musculus transformation related protein 53 inducible nuclear protein 1 (Trp53inp1), mRNA.
0.002094	4.67E-05	1.6	up	Sult1a1	20887	Mus musculus sulfotransferase family 1A, phenol-preferring, member 1 (Sult1a1), mRNA.
0.007288	3.81E-04	1.6	up	Tagln2	21346	Mus musculus transgelin 2 (Tagln2), mRNA.
0.036199	0.00447	1.6	up	Anxa5	11747	Mus musculus annexin A5 (Anxa5), mRNA.
0.003866	1.37E-04	1.6	up	Anxa3	11745	Mus musculus annexin A3 (Anxa3), mRNA.
0.001529	2.77E-05	1.6	up	Tes	21753	Mus musculus testis derived transcript (Tes), transcript variant 1, mRNA.
4.80E-04	4.09E-06	1.6	up	Icam2	15896	Mus musculus intercellular adhesion molecule 2 (Icam2), mRNA.
0.011891	8.01E-04	1.6	up	C1qc	12262	Mus musculus complement component 1, q subcomponent, C chain (C1qc), mRNA.
4.22E-05	6.53E-08	1.6	up	Bax	12028	Mus musculus Bcl2-associated X protein (Bax), mRNA.
0.010554	6.62E-04	1.6	up	Nrbp2	223649	Mus musculus nuclear receptor binding protein 2 (Nrbp2), mRNA.
0.004473	1.71E-04	1.6	up	Ptp4a2	19244	Mus musculus protein tyrosine phosphatase 4a2 (Ptp4a2), mRNA.
0.022049	0.002137	1.6	up	2810402K13Rik		
4.77E-04	4.02E-06	1.6	up	Ttpa	50500	Mus musculus tocopherol (alpha) transfer protein (Ttpa), mRNA.
0.003216	9.64E-05	1.6	up	Vwf	22371	Mus musculus Von Willebrand factor homolog (Vwf), mRNA.

2.21E-04	1.14E-06	1.6	up	Mustn1	66175	Mus musculus musculoskeletal, embryonic nuclear protein 1 (Mustn1), mRNA.
0.005162	2.14E-04	1.6	up	Tbx3	21386	Mus musculus T-box 3 (Tbx3), transcript variant 1, mRNA.
0.003503	1.13E-04	1.6	up	scl0001379.1_70		
8.88E-04	1.14E-05	1.6	up	Prlr	19116	Mus musculus prolactin receptor (Prlr), mRNA.
7.75E-04	9.28E-06	1.6	up	Hp	15439	Mus musculus haptoglobin (Hp), mRNA.
0.044241	0.006033	1.6	up	Arl4a	11861	Mus musculus ADP-ribosylation factor-like 4A (Arl4a), transcript variant 1, mRNA.
0.008156	4.52E-04	1.6	up	Ptp4a2	19244	Mus musculus protein tyrosine phosphatase 4a2 (Ptp4a2), mRNA.
2.54E-04	1.48E-06	1.6	up	Itih3		
0.010441	6.50E-04	1.6	up	2810026P18Rik		
0.004971	2.02E-04	1.6	up	Eltd1	170757	Mus musculus EGF, latrophilin seven transmembrane domain containing 1 (Eltd1), mRNA.
0.003861	1.37E-04	1.6	up	Itih4	16427	Mus musculus inter alpha-trypsin inhibitor, heavy chain 4 (Itih4), mRNA.
0.034179	0.004125	1.6	up	Slc35c1	228368	Mus musculus solute carrier family 35, member C1 (Slc35c1), transcript variant 2, mRNA.
0.003061	8.68E-05	1.6	up	Plac8	231507	Mus musculus placenta-specific 8 (Plac8), mRNA.
0.01556	0.001238	1.6	up	Arhgef3	71704	Mus musculus Rho guanine nucleotide exchange factor (GEF) 3 (Arhgef3), mRNA.
0.010035	6.14E-04	1.6	up	Lum	17022	Mus musculus lumican (Lum), mRNA.
0.001523	2.73E-05	1.6	up	Fos	14281	Mus musculus FBJ osteosarcoma oncogene (Fos), mRNA.
0.006792	3.38E-04	1.6	up	Map3k1	26401	Mus musculus mitogen-activated protein kinase kinase kinase 1 (Map3k1), mRNA.
3.19E-04	2.23E-06	1.5	up	Vim	22352	Mus musculus vimentin (Vim), mRNA.
0.001028	1.44E-05	1.5	up	Agt	11606	Mus musculus angiotensinogen (serpin peptidase inhibitor, clade A, member 8) (Agt), mRNA.
0.001599	2.97E-05	1.5	up	Abcd2	26874	Mus musculus ATP-binding cassette, sub-family D (ALD), member 2 (Abcd2), mRNA.
0.003803	1.32E-04	1.5	up	Csrp3	13009	Mus musculus cysteine and glycine-rich protein 3 (Csrp3), mRNA.
0.003527	1.15E-04	1.5	up	Grn	14824	Mus musculus granulin (Grn), mRNA.

0.003479	1.10E-04	1.5	up	Tes	21753	Mus musculus testis derived transcript (Tes), transcript variant 1, mRNA.
4.66E-04	3.88E-06	1.5	up	LOC100046883	100046883	PREDICTED: Mus musculus similar to CKLF-like MARVEL transmembrane domain containing 3 (LOC100046883), mRNA.
0.001165	1.76E-05	1.5	up	Ppap2c	50784	Mus musculus phosphatidic acid phosphatase type 2c (Ppap2c), mRNA.
0.007871	4.30E-04	1.5	up	Ang	11727	Mus musculus angiogenin, ribonuclease, RNase A family, 5 (Ang), mRNA.
0.006813	3.43E-04	1.5	up	Zfp36l1	12192	Mus musculus zinc finger protein 36, C3H type-like 1 (Zfp36l1), mRNA.
0.027534	0.003003	1.5	up	382044	382044	Mus musculus predicted gene, 382044 (382044), mRNA.
0.014197	0.001079	1.5	up	Chi3l3		
0.020106	0.001844	1.5	up	Col4a2	12827	Mus musculus collagen, type IV, alpha 2 (Col4a2), mRNA.
2.25E-05	2.25E-08	1.5	up	Ramp1	51801	Mus musculus receptor (calcitonin) activity modifying protein 1 (Ramp1), mRNA.
0.013437	9.83E-04	1.5	up	Ppp2r2d	52432	Mus musculus protein phosphatase 2, regulatory subunit B, delta isoform (Ppp2r2d), mRNA.
0.012709	9.01E-04	1.5	up	Dusp6	67603	Mus musculus dual specificity phosphatase 6 (Dusp6), mRNA.
8.88E-04	1.13E-05	1.5	up	Serpina11	380780	PREDICTED: Mus musculus serine (or cysteine) peptidase inhibitor, clade A (alpha-1 antiproteinase, antitrypsin), member 11 (Serpina11), mRNA.
7.55E-04	8.81E-06	1.5	up	Lcat	16816	Mus musculus lecithin cholesterol acyltransferase (Lcat), mRNA.
0.00183	3.63E-05	1.5	up	Ccng1	12450	Mus musculus cyclin G1 (Ccng1), mRNA.
0.013707	0.001017	1.5	up	1190002N15Rik		
0.011988	8.10E-04	1.5	up	Slc25a33	70556	Mus musculus solute carrier family 25, member 33 (Slc25a33), mRNA.
0.006692	3.30E-04	1.5	up	1190002N15Rik	68861	Mus musculus RIKEN cDNA 1190002N15 gene (1190002N15Rik), mRNA.
0.019817	0.001801	1.5	up	Arl4a	11861	Mus musculus ADP-ribosylation factor-like 4A (Arl4a), transcript variant 1, mRNA.
0.01896	0.001684	1.5	up	Olfr707	194433	Mus musculus olfactory receptor 707 (Olfr707), mRNA.
3.04E-04	2.04E-06	1.5	up	Nicn1	66257	Mus musculus nicolin 1 (Nicn1), mRNA.
0.005201	2.17E-04	1.5	up	C4b	12268	Mus musculus complement component 4B (Childo blood group) (C4b), mRNA. XM_921663 XM_921673 XM_921676 XM_921678
0.005818	2.60E-04	1.5	up	Gucy1a3	60596	Mus musculus guanylate cyclase 1, soluble, alpha 3 (Gucy1a3), mRNA.
0.003065	8.81E-05	1.5	up	Ccng1	12450	Mus musculus cyclin G1 (Ccng1), mRNA.

0.004904	1.98E-04	1.5	up	Ear2	13587	Mus musculus eosinophil-associated, ribonuclease A family, member 2 (Ear2), mRNA.
0.004157	1.54E-04	1.5	up	Avpr1a	54140	Mus musculus arginine vasopressin receptor 1A (Avpr1a), mRNA.
0.004165	1.55E-04	1.5	up	Lcat	16816	Mus musculus lecithin cholesterol acyltransferase (Lcat), mRNA.
0.00944	5.63E-04	1.5	up	Slc41a2	338365	Mus musculus solute carrier family 41, member 2 (Slc41a2), mRNA.
4.48E-04	3.68E-06	1.5	up	Armcx2	67416	Mus musculus armadillo repeat containing, X-linked 2 (Armcx2), mRNA.
0.035187	0.004298	1.5	up	Igl-V1		
0.00474	1.86E-04	1.5	up	Raet1b	19369	Mus musculus retinoic acid early transcript beta (Raet1b), mRNA.
0.003446	1.08E-04	1.5	up	Ilvbl	216136	Mus musculus ilvB (bacterial acetolactate synthase)-like (Ilvbl), mRNA.
0.003843	1.36E-04	1.5	up	LOC100045680	100045680	PREDICTED: Mus musculus similar to complement C4 (LOC100045680), mRNA.
0.023903	0.002421	1.5	up	A630082K20Rik		
0.003381	1.05E-04	1.5	up	Raet1b	19369	Mus musculus retinoic acid early transcript beta (Raet1b), mRNA.
2.54E-04	1.47E-06	1.5	up	Pros1	19128	Mus musculus protein S (alpha) (Pros1), mRNA.
0.006189	2.89E-04	1.5	up	Cd93	17064	Mus musculus CD93 antigen (Cd93), mRNA.
9.80E-04	1.35E-05	1.5	up	Sema3f	20350	Mus musculus sema domain, immunoglobulin domain (Ig), short basic domain, secreted, (semaphorin) 3F (Sema3f), mRNA.
0.001169	1.78E-05	8.3	down	Cyp4a12b	13118	Mus musculus cytochrome P450, family 4, subfamily a, polypeptide 12B (Cyp4a12b), mRNA.
0.009669	5.81E-04	7.7	down	Elovl3		
5.21E-04	4.80E-06	6.4	down	Thrsp	21835	Mus musculus thyroid hormone responsive SPOT14 homolog (Rattus) (Thrsp), mRNA.
0.00368	1.25E-04	5.7	down	LOC236060		
6.29E-04	6.63E-06	4.9	down	LOC100044164	100044164	PREDICTED: Mus musculus hypothetical protein LOC100044164 (LOC100044164), mRNA.
0.00813	4.50E-04	4.5	down	Cyp4a12a	277753	Mus musculus cytochrome P450, family 4, subfamily a, polypeptide 12a (Cyp4a12a), mRNA.
9.61E-04	1.29E-05	4.5	down	Cyp2a5	13087	Mus musculus cytochrome P450, family 2, subfamily a, polypeptide 5 (Cyp2a5), mRNA.
6.16E-05	1.09E-07	4.5	down	EG241041	241041	Mus musculus predicted gene, EG241041 (EG241041), non-coding RNA.

0.003859	1.37E-04	4.4	down	Elovl3	12686	Mus musculus elongation of very long chain fatty acids (FEN1/Elo2, SUR4/Elo3, yeast)-like 3 (Elovl3), mRNA.
3.29E-04	2.45E-06	4.2	down	EG241041	241041	Mus musculus predicted gene, EG241041 (EG241041), non-coding RNA.
3.00E-04	2.00E-06	3.9	down	Cyp2a5	13087	Mus musculus cytochrome P450, family 2, subfamily a, polypeptide 5 (Cyp2a5), mRNA.
0.002592	6.64E-05	3.8	down	Mup4	17843	Mus musculus major urinary protein 4 (Mup4), mRNA.
2.96E-04	1.95E-06	3.7	down	Elovl6	170439	Mus musculus ELOVL family member 6, elongation of long chain fatty acids (yeast) (Elovl6), mRNA.
0.018573	0.001634	3.5	down	OTTMUSG00000007485	381530	Mus musculus predicted gene, OTTMUSG00000007485 (OTTMUSG00000007485), mRNA.
0.002696	7.08E-05	3.4	down	Serpina4-ps1	321018	Mus musculus serine (or cysteine) peptidase inhibitor, clade A, member 4, pseudogene 1 (Serpina4-ps1), non-coding RNA.
3.27E-04	2.38E-06	3.4	down	Hes6	55927	Mus musculus hairy and enhancer of split 6 (Drosophila) (Hes6), mRNA.
1.58E-04	6.72E-07	3.3	down	Aacs	78894	Mus musculus acetoacetyl-CoA synthetase (Aacs), mRNA.
1.25E-04	4.82E-07	3.3	down	LOC677317	677317	PREDICTED: Mus musculus similar to Mod1 protein, transcript variant 4 (LOC677317), mRNA.
0.003065	8.86E-05	3.3	down	Cyp2a5	13087	Mus musculus cytochrome P450, family 2, subfamily a, polypeptide 5 (Cyp2a5), mRNA.
0.00368	1.26E-04	3.3	down	Nudt7		
2.10E-04	1.05E-06	3.1	down	Acly	104112	Mus musculus ATP citrate lyase (Acly), mRNA.
0.004647	1.81E-04	3.1	down	Igh-6		
0.002564	6.49E-05	3.0	down	Hes6	55927	Mus musculus hairy and enhancer of split 6 (Drosophila) (Hes6), mRNA.
1.09E-04	3.22E-07	3.0	down	Mod1	17436	Mus musculus malic enzyme, supernatant (Mod1), mRNA.
0.001268	2.05E-05	3.0	down	Hes6	55927	Mus musculus hairy and enhancer of split 6 (Drosophila) (Hes6), mRNA.
0.001382	2.32E-05	2.9	down	LOC384022		
7.44E-06	4.47E-09	2.9	down	Olig1	50914	Mus musculus oligodendrocyte transcription factor 1 (Olig1), mRNA.
5.21E-04	4.79E-06	2.8	down	Acss2	60525	Mus musculus acyl-CoA synthetase short-chain family member 2 (Acss2), mRNA.
0.006821	3.45E-04	2.8	down	Igh-6		

6.29E-04	6.46E-06	2.8	down	Acacb		
0.003063	8.73E-05	2.7	down	Sucnr1	84112	Mus musculus succinate receptor 1 (Sucnr1), mRNA.
3.93E-05	5.14E-08	2.7	down	Klk1b4	18048	Mus musculus kallikrein 1-related pepidase b4 (Klk1b4), mRNA.
6.94E-04	7.90E-06	2.7	down	Mvd	192156	Mus musculus mevalonate (diphospho) decarboxylase (Mvd), mRNA.
5.22E-04	4.84E-06	2.7	down	Pnpla5		
6.39E-04	6.99E-06	2.6	down	Nudt7	67528	Mus musculus nudix (nucleoside diphosphate linked moiety X)-type motif 7 (Nudt7), transcript variant 2, mRNA.
0.00183	3.63E-05	2.6	down	Fkbp11	66120	Mus musculus FK506 binding protein 11 (Fkbp11), mRNA.
0.001971	4.25E-05	2.5	down	Srd5a1		
8.28E-04	1.01E-05	2.5	down	LOC100043671	100043671	PREDICTED: Mus musculus hypothetical protein LOC100043671 (LOC100043671), mRNA.
1.07E-04	3.12E-07	2.5	down	Klk1b4	18048	Mus musculus kallikrein 1-related pepidase b4 (Klk1b4), mRNA.
0.003745	1.29E-04	2.5	down	Rdh16	19683	Mus musculus retinol dehydrogenase 16 (Rdh16), mRNA.
0.003311	1.00E-04	2.5	down	Nudt7	67528	Mus musculus nudix (nucleoside diphosphate linked moiety X)-type motif 7 (Nudt7), transcript variant 1, mRNA.
0.00253	6.35E-05	2.5	down	2810439F02Rik	72747	Mus musculus RIKEN cDNA 2810439F02 gene (2810439F02Rik), mRNA.
0.00714	3.69E-04	2.4	down	Chrna4	11438	Mus musculus cholinergic receptor, nicotinic, alpha polypeptide 4 (Chrna4), mRNA.
4.12E-04	3.22E-06	2.4	down	Hsp105		
0.00215	4.87E-05	2.4	down	Rdh11		
0.044314	0.006048	2.4	down	OTTMUSG00000000231	381531	Mus musculus predicted gene, OTTMUSG00000000231 (OTTMUSG00000000231), mRNA.
0.007115	3.66E-04	2.4	down	EG13909	13909	Mus musculus predicted gene, EG13909 (EG13909), mRNA.
0.001165	1.77E-05	2.3	down	Pklr	18770	Mus musculus pyruvate kinase liver and red blood cell (Pklr), mRNA.
4.80E-04	4.07E-06	2.3	down	Fasn	14104	Mus musculus fatty acid synthase (Fasn), mRNA.
0.001145	1.69E-05	2.3	down	Gstp1	14870	Mus musculus glutathione S-transferase, pi 1 (Gstp1), mRNA.
0.004765	1.88E-04	2.3	down	Pnpla3	116939	Mus musculus patatin-like phospholipase domain containing 3 (Pnpla3), mRNA.
0.007169	3.72E-04	2.3	down	Hamp2	66438	Mus musculus hepcidin antimicrobial peptide 2 (Hamp2), mRNA.

0.00215	4.87E-05	2.3	down	Ntrk2	18212	Mus musculus neurotrophic tyrosine kinase, receptor, type 2 (Ntrk2), transcript variant 1, mRNA.
0.009228	5.44E-04	2.3	down	LOC245892		
0.003043	8.54E-05	2.3	down	H2-Ab1	14961	Mus musculus histocompatibility 2, class II antigen A, beta 1 (H2-Ab1), mRNA.
0.006522	3.14E-04	2.3	down	2810439F02Rik	72747	Mus musculus RIKEN cDNA 2810439F02 gene (2810439F02Rik), mRNA.
1.60E-04	7.08E-07	2.2	down	Sgk2	27219	Mus musculus serum/glucocorticoid regulated kinase 2 (Sgk2), mRNA.
0.003124	9.25E-05	2.2	down	Rdh11	17252	Mus musculus retinol dehydrogenase 11 (Rdh11), mRNA.
0.00279	7.50E-05	2.2	down	G6pdx	14381	Mus musculus glucose-6-phosphate dehydrogenase X-linked (G6pdx), mRNA.
0.01229	8.47E-04	2.2	down	LOC666559	666559	PREDICTED: Mus musculus similar to farnesyl pyrophosphate synthase (LOC666559), misc RNA.
0.0035	1.12E-04	2.2	down	Fdps	110196	Mus musculus farnesyl diphosphate synthetase (Fdps), mRNA.
0.005425	2.31E-04	2.2	down	Aqp8	11833	Mus musculus aquaporin 8 (Aqp8), mRNA.
4.94E-04	4.23E-06	2.2	down	Sgk2	27219	Mus musculus serum/glucocorticoid regulated kinase 2 (Sgk2), mRNA.
0.01342	9.80E-04	2.2	down	Chrna4	11438	Mus musculus cholinergic receptor, nicotinic, alpha polypeptide 4 (Chrna4), mRNA.
0.004075	1.50E-04	2.2	down	LOC641240	641240	PREDICTED: Mus musculus similar to MHC class II antigen beta chain (LOC641240), mRNA.
0.001396	2.39E-05	2.2	down	Cyp2d40	71754	Mus musculus cytochrome P450, family 2, subfamily d, polypeptide 40 (Cyp2d40), mRNA.
0.001891	3.97E-05	2.2	down	1500017E21Rik		
0.020719	0.001929	2.1	down	Cxcl9	17329	Mus musculus chemokine (C-X-C motif) ligand 9 (Cxcl9), mRNA.
0.010993	7.03E-04	2.1	down	Mid1ip1	68041	Mus musculus Mid1 interacting protein 1 (gastrulation specific G12-like (zebrafish)) (Mid1ip1), mRNA.
0.004507	1.73E-04	2.1	down	Bdh2	69772	Mus musculus 3-hydroxybutyrate dehydrogenase, type 2 (Bdh2), mRNA.
0.001204	1.87E-05	2.1	down	LOC100046163	100046163	PREDICTED: Mus musculus similar to Nme6 protein (LOC100046163), mRNA.
0.001599	2.95E-05	2.1	down	H2-Eb1	14969	Mus musculus histocompatibility 2, class II antigen E beta (H2-Eb1), mRNA.
0.003479	1.11E-04	2.1	down	Gstp1	14870	Mus musculus glutathione S-transferase, pi 1 (Gstp1), mRNA.

0.002167	4.96E-05	2.1	down	Ddah1		
1.25E-04	4.88E-07	2.1	down	Reck		
0.01145	7.49E-04	2.1	down	Acpp	56318	Mus musculus acid phosphatase, prostate (Acpp), transcript variant 1, mRNA.
0.002258	5.33E-05	2.1	down	Tlcd2	380712	Mus musculus TLC domain containing 2 (Tlcd2), mRNA.
0.001834	3.66E-05	2.1	down	Gna14	14675	Mus musculus guanine nucleotide binding protein, alpha 14 (Gna14), mRNA.
0.005649	2.47E-04	2.0	down	Aqp8	11833	Mus musculus aquaporin 8 (Aqp8), mRNA.
0.012539	8.84E-04	2.0	down	Gpam	14732	Mus musculus glycerol-3-phosphate acyltransferase, mitochondrial (Gpam), nuclear gene encoding mitochondrial protein, mRNA.
0.001499	2.67E-05	2.0	down	Igfbp2	16008	Mus musculus insulin-like growth factor binding protein 2 (Igfbp2), mRNA.
0.001214	1.91E-05	2.0	down	Gstp1	14870	Mus musculus glutathione S-transferase, pi 1 (Gstp1), mRNA.
2.68E-04	1.63E-06	2.0	down	LOC100044756	100044756	PREDICTED: Mus musculus similar to PX domain-containing protein kinase-like protein (Modulator of Na,K-ATPase) (MONaKA) (LOC100044756), mRNA.
0.020611	0.001914	2.0	down	Car3	12350	Mus musculus carbonic anhydrase 3 (Car3), mRNA.
0.002151	4.90E-05	2.0	down	Pklr	18770	Mus musculus pyruvate kinase liver and red blood cell (Pklr), mRNA.
1.94E-05	1.82E-08	2.0	down	Xkr9	381246	Mus musculus X Kell blood group precursor related family member 9 homolog (Xkr9), mRNA.
0.015292	0.001203	2.0	down	Alas2		
0.008697	4.96E-04	2.0	down	Per2	18627	Mus musculus period homolog 2 (Drosophila) (Per2), mRNA.
0.001068	1.52E-05	2.0	down	Scap	235623	Mus musculus SREBF chaperone (Scap), mRNA.
0.002756	7.35E-05	2.0	down	EG13909	13909	Mus musculus predicted gene, EG13909 (EG13909), mRNA.
2.04E-04	9.90E-07	1.9	down	Cyp4v3	102294	Mus musculus cytochrome P450, family 4, subfamily v, polypeptide 3 (Cyp4v3), mRNA.
0.006448	3.05E-04	1.9	down	EG13909	13909	Mus musculus predicted gene, EG13909 (EG13909), mRNA.
0.028296	0.003126	1.9	down	Keg1	64697	Mus musculus kidney expressed gene 1 (Keg1), mRNA.
0.001796	3.49E-05	1.9	down	A530050D06Rik	104816	Mus musculus RIKEN cDNA A530050D06 gene (A530050D06Rik), mRNA.
1.84E-05	1.47E-08	1.9	down	LOC100046163	100046163	PREDICTED: Mus musculus similar to Nme6 protein (LOC100046163), mRNA.
2.62E-04	1.56E-06	1.9	down	Acat2	110460	Mus musculus acetyl-Coenzyme A acetyltransferase 2 (Acat2), mRNA.

0.007169	3.72E-04	1.9	down	Dio1	13370	Mus musculus deiodinase, iodothyronine, type I (Dio1), mRNA.
0.001529	2.77E-05	1.9	down	Tlcd2	380712	Mus musculus TLC domain containing 2 (Tlcd2), mRNA.
9.80E-04	1.35E-05	1.9	down	Pstpip2	19201	Mus musculus proline-serine-threonine phosphatase-interacting protein 2 (Pstpip2), mRNA.
0.007117	3.67E-04	1.9	down	Fabp5	16592	Mus musculus fatty acid binding protein 5, epidermal (Fabp5), mRNA.
2.01E-04	9.56E-07	1.9	down	LOC245069		
0.002592	6.64E-05	1.9	down	Sc5d		
0.021797	0.002092	1.9	down	Nsdhl	18194	Mus musculus NAD(P) dependent steroid dehydrogenase-like (Nsdhl), mRNA.
0.00646	3.08E-04	1.9	down	Insig1	231070	Mus musculus insulin induced gene 1 (Insig1), mRNA.
0.001888	3.90E-05	1.9	down	Nudt7		
0.005425	2.31E-04	1.9	down	Acss2	60525	Mus musculus acyl-CoA synthetase short-chain family member 2 (Acss2), mRNA.
0.001504	2.68E-05	1.9	down	Igfbp2	16008	Mus musculus insulin-like growth factor binding protein 2 (Igfbp2), mRNA.
0.002046	4.47E-05	1.9	down	Pdzk1ip1	67182	Mus musculus PDZK1 interacting protein 1 (Pdzk1ip1), mRNA.
1.30E-04	5.28E-07	1.9	down	Gm2a	14667	Mus musculus GM2 ganglioside activator protein (Gm2a), mRNA.
1.12E-04	3.68E-07	1.9	down	Cyp2u1	71519	Mus musculus cytochrome P450, family 2, subfamily u, polypeptide 1 (Cyp2u1), mRNA.
0.014163	0.001075	1.8	down	Cyp51	13121	Mus musculus cytochrome P450, family 51 (Cyp51), mRNA.
0.028839	0.003223	1.8	down	Nsdhl	18194	Mus musculus NAD(P) dependent steroid dehydrogenase-like (Nsdhl), mRNA.
5.22E-04	4.93E-06	1.8	down	Hmgcr	15357	Mus musculus 3-hydroxy-3-methylglutaryl-Coenzyme A reductase (Hmgcr), mRNA.
0.003067	8.93E-05	1.8	down	H2-Ab1	14961	Mus musculus histocompatibility 2, class II antigen A, beta 1 (H2-Ab1), mRNA.
0.004643	1.81E-04	1.8	down	Ak2	11637	Mus musculus adenylate kinase 2 (Ak2), transcript variant 1, mRNA.
0.019667	0.001779	1.8	down	Tuba6		
0.005025	2.05E-04	1.8	down	Sdf2l1	64136	Mus musculus stromal cell-derived factor 2-like 1 (Sdf2l1), mRNA.
0.003527	1.15E-04	1.8	down	Insig1	231070	Mus musculus insulin induced gene 1 (Insig1), mRNA.
0.004881	1.95E-04	1.8	down	Cyp2f2	13107	Mus musculus cytochrome P450, family 2, subfamily f, polypeptide 2 (Cyp2f2), mRNA.

0.001842	3.70E-05	1.8	down	Per2	18627	Mus musculus period homolog 2 (Drosophila) (Per2), mRNA.
2.54E-04	1.50E-06	1.8	down	Gstm6	14867	Mus musculus glutathione S-transferase, mu 6 (Gstm6), mRNA.
0.008169	4.54E-04	1.8	down	Cyp1a2	13077	Mus musculus cytochrome P450, family 1, subfamily a, polypeptide 2 (Cyp1a2), mRNA.
3.24E-04	2.28E-06	1.8	down	Mrap	77037	Mus musculus melanocortin 2 receptor accessory protein (Mrap), mRNA.
9.58E-04	1.27E-05	1.8	down	Pnpla5	75772	Mus musculus patatin-like phospholipase domain containing 5 (Pnpla5), mRNA.
2.50E-04	1.43E-06	1.8	down	Gstm6	14867	Mus musculus glutathione S-transferase, mu 6 (Gstm6), mRNA.
9.16E-05	2.16E-07	1.8	down	Tbcel	272589	Mus musculus tubulin folding cofactor E-like (Tbcel), mRNA.
6.29E-04	6.68E-06	1.8	down	Klk1b4	18048	Mus musculus kallikrein 1-related pepidase b4 (Klk1b4), mRNA.
0.026541	0.002853	1.8	down	Cd74	16149	Mus musculus CD74 antigen (invariant polypeptide of major histocompatibility complex, class II antigen-associated) (Cd74), transcript variant 2, mRNA.
0.026928	0.002915	1.8	down	Lss	16987	Mus musculus lanosterol synthase (Lss), mRNA.
0.003535	1.16E-04	1.8	down	2310045A20Rik	231238	Mus musculus RIKEN cDNA 2310045A20 gene (2310045A20Rik), mRNA.
0.01719	0.001425	1.8	down	LOC100040592	100040592	PREDICTED: Mus musculus similar to Hmgcs1 protein, transcript variant 1 (LOC100040592), mRNA.
0.006527	3.16E-04	1.8	down	Acaa1b	235674	Mus musculus acetyl-Coenzyme A acyltransferase 1B (Acaa1b), mRNA.
0.018315	0.001591	1.8	down	H2-Aa	14960	Mus musculus histocompatibility 2, class II antigen A, alpha (H2-Aa), mRNA.
0.005012	2.04E-04	1.8	down	Pgd		
0.043749	0.005935	1.8	down	Ugt2b1	71773	Mus musculus UDP glucuronosyltransferase 2 family, polypeptide B1 (Ugt2b1), mRNA.
5.72E-04	5.64E-06	1.8	down	Gstm6		
0.005794	2.58E-04	1.8	down	Dhcr7	13360	Mus musculus 7-dehydrocholesterol reductase (Dhcr7), mRNA.
0.005576	2.42E-04	1.8	down	Dio1	13370	Mus musculus deiodinase, iodothyronine, type I (Dio1), mRNA.
0.004745	1.86E-04	1.8	down	C9	12279	Mus musculus complement component 9 (C9), mRNA.
0.034864	0.004239	1.8	down	Upp2	76654	Mus musculus uridine phosphorylase 2 (Upp2), mRNA.
9.58E-04	1.28E-05	1.7	down	Gale	74246	Mus musculus galactose-4-epimerase, UDP (Gale), mRNA.
0.02873	0.003198	1.7	down	LOC669658	669658	PREDICTED: Mus musculus similar to melanoma antigen (LOC669658), mRNA.

0.014064	0.001062	1.7	down	Susd4	96935	Mus musculus sushi domain containing 4 (Susd4), mRNA.
1.12E-04	3.68E-07	1.7	down	Abhd5	67469	Mus musculus abhydrolase domain containing 5 (Abhd5), mRNA.
1.94E-05	1.85E-08	1.7	down	Rpia	19895	Mus musculus ribose 5-phosphate isomerase A (Rpia), mRNA.
1.21E-04	4.37E-07	1.7	down	Reck	53614	Mus musculus reversion-inducing-cysteine-rich protein with kazal motifs (Reck), mRNA.
0.002453	6.04E-05	1.7	down	Xkr9	381246	Mus musculus X Kell blood group precursor related family member 9 homolog (Xkr9), mRNA.
0.040478	0.005281	1.7	down	Creld2	76737	Mus musculus cysteine-rich with EGF-like domains 2 (Creld2), mRNA.
0.023091	0.002286	1.7	down	Cyp1a2	13077	Mus musculus cytochrome P450, family 1, subfamily a, polypeptide 2 (Cyp1a2), mRNA.
0.0126	8.91E-04	1.7	down	Sc4mol	66234	Mus musculus sterol-C4-methyl oxidase-like (Sc4mol), mRNA.
0.005755	2.54E-04	1.7	down	Aox3	71724	Mus musculus aldehyde oxidase 3 (Aox3), mRNA.
6.31E-04	6.74E-06	1.7	down	Cd8b		
5.11E-04	4.56E-06	1.7	down	Paox	212503	Mus musculus polyamine oxidase (exo-N4-amino) (Paox), mRNA.
0.015148	0.001184	1.7	down	Krt23	94179	Mus musculus keratin 23 (Krt23), mRNA.
0.039309	0.005063	1.7	down	Cd74	16149	Mus musculus CD74 antigen (invariant polypeptide of major histocompatibility complex, class II antigen-associated) (Cd74), transcript variant 1, mRNA.
0.014932	0.001157	1.7	down	Srebf1	20787	Mus musculus sterol regulatory element binding factor 1 (Srebf1), mRNA.
0.016346	0.001328	1.7	down	2010305C02Rik		
0.00282	7.62E-05	1.7	down	5730472N09Rik	108958	Mus musculus RIKEN cDNA 5730472N09 gene (5730472N09Rik), mRNA.
0.002593	6.65E-05	1.7	down	Cyb5r3	109754	Mus musculus cytochrome b5 reductase 3 (Cyb5r3), mRNA.
9.11E-04	1.18E-05	1.7	down	Rdbp	27632	Mus musculus RD RNA-binding protein (Rdbp), transcript variant 3, mRNA.
0.013467	9.88E-04	1.7	down	S100a10	20194	Mus musculus S100 calcium binding protein A10 (calpactin) (S100a10), mRNA.
1.12E-04	3.77E-07	1.7	down	Tssc1	380752	Mus musculus tumor suppressing subtransferable candidate 1 (Tssc1), mRNA.
0.00714	3.69E-04	1.7	down	Cyp2d9	13105	Mus musculus cytochrome P450, family 2, subfamily d, polypeptide 9 (Cyp2d9), mRNA.
0.001919	4.06E-05	1.7	down	Pdhb	68263	Mus musculus pyruvate dehydrogenase (lipoamide) beta (Pdhb), mRNA.

0.004507	1.73E-04	1.7	down	Gamt	14431	Mus musculus guanidinoacetate methyltransferase (Gamt), mRNA.
2.98E-04	1.97E-06	1.7	down	2900024O10Rik	72852	Mus musculus RIKEN cDNA 2900024O10 gene (2900024O10Rik), mRNA.
0.005701	2.51E-04	1.6	down	Ugt3a1	105887	Mus musculus UDP glycosyltransferases 3 family, polypeptide A1 (Ugt3a1), mRNA.
0.001384	2.34E-05	1.6	down	Paox	212503	Mus musculus polyamine oxidase (exo-N4-amino) (Paox), mRNA.
5.21E-04	4.79E-06	1.6	down	Cyp2a5	13087	Mus musculus cytochrome P450, family 2, subfamily a, polypeptide 5 (Cyp2a5), mRNA.
0.030371	0.003445	1.6	down	H2-Ab1	14961	Mus musculus histocompatibility 2, class II antigen A, beta 1 (H2-Ab1), mRNA.
0.009375	5.58E-04	1.6	down	Edem2	108687	Mus musculus ER degradation enhancer, mannosidase alpha-like 2 (Edem2), mRNA.
0.032973	0.003918	1.6	down	Dclk3	245038	Mus musculus doublecortin-like kinase 3 (Dclk3), mRNA.
0.015692	0.001253	1.6	down	Rassf3	192678	Mus musculus Ras association (RalGDS/AF-6) domain family member 3 (Rassf3), mRNA.
0.009041	5.25E-04	1.6	down	Cs	12974	Mus musculus citrate synthase (Cs), nuclear gene encoding mitochondrial protein, mRNA.
0.004125	1.52E-04	1.6	down	Pdhb	68263	Mus musculus pyruvate dehydrogenase (lipoamide) beta (Pdhb), mRNA.
0.021487	0.00203	1.6	down	Gpr146	80290	Mus musculus G protein-coupled receptor 146 (Gpr146), transcript variant 2, mRNA.
5.07E-04	4.45E-06	1.6	down	5730469M10Rik	70564	Mus musculus RIKEN cDNA 5730469M10 gene (5730469M10Rik), mRNA.
0.00338	1.05E-04	1.6	down	Ero1lb	67475	Mus musculus ERO1-like beta (S. cerevisiae) (Ero1lb), mRNA.
0.003816	1.34E-04	1.6	down	Paox	212503	Mus musculus polyamine oxidase (exo-N4-amino) (Paox), mRNA.
0.001822	3.57E-05	1.6	down	Ak2	11637	Mus musculus adenylate kinase 2 (Ak2), transcript variant 2, mRNA.
2.90E-04	1.83E-06	1.6	down	AU018778	234564	Mus musculus expressed sequence AU018778 (AU018778), mRNA.
0.004256	1.60E-04	1.6	down	Dak	225913	Mus musculus dihydroxyacetone kinase 2 homolog (yeast) (Dak), mRNA.
8.31E-04	1.03E-05	1.6	down	Rnf103	22644	Mus musculus ring finger protein 103 (Rnf103), mRNA.
0.007823	4.27E-04	1.6	down	A730017D01Rik		
0.005564	2.41E-04	1.6	down	Pik3c2g		
0.004507	1.73E-04	1.6	down	N6amt2	68043	Mus musculus N-6 adenine-specific DNA methyltransferase 2 (putative) (N6amt2), mRNA.

0.037607	0.004744	1.6	down	Creld2	76737	Mus musculus cysteine-rich with EGF-like domains 2 (Creld2), mRNA.
0.009505	5.68E-04	1.6	down	Cyb5r3	109754	Mus musculus cytochrome b5 reductase 3 (Cyb5r3), mRNA.
0.007947	4.35E-04	1.6	down	Aldh1a7	26358	Mus musculus aldehyde dehydrogenase family 1, subfamily A7 (Aldh1a7), mRNA.
0.001856	3.77E-05	1.6	down	Sort1	20661	Mus musculus sortilin 1 (Sort1), mRNA.
0.018411	0.001604	1.6	down	1810005K13Rik		
0.00899	5.18E-04	1.6	down	Gpr110		
0.00508	2.09E-04	1.6	down	Ralgps1		
0.018729	0.001656	1.6	down	Hopx	74318	Mus musculus HOP homeobox (Hopx), mRNA.
0.004976	2.03E-04	1.6	down	Mlxipl	58805	Mus musculus MLX interacting protein-like (Mlxipl), mRNA.
0.008408	4.74E-04	1.6	down	Oprs1	18391	Mus musculus opioid receptor, sigma 1 (Oprs1), mRNA.
7.47E-04	8.69E-06	1.6	down	Thnsl2	232078	Mus musculus threonine synthase-like 2 (bacterial) (Thnsl2), transcript variant 2, mRNA.
0.003374	1.04E-04	1.6	down	Dhrs7b	216820	Mus musculus dehydrogenase/reductase (SDR family) member 7B (Dhrs7b), mRNA.
0.033284	0.00399	1.6	down	LOC382092		
0.006527	3.17E-04	1.6	down	Pcsk9	100102	Mus musculus proprotein convertase subtilisin/kexin type 9 (Pcsk9), mRNA.
0.001596	2.93E-05	1.6	down	Fads2		
6.39E-04	6.98E-06	1.6	down	Chordc1	66917	Mus musculus cysteine and histidine-rich domain (CHORD)-containing, zinc-binding protein 1 (Chordc1), mRNA.
0.005946	2.70E-04	1.6	down	Ak2	11637	Mus musculus adenylate kinase 2 (Ak2), transcript variant 2, mRNA.
1.12E-04	3.57E-07	1.6	down	Extl1		
0.01254	8.84E-04	1.6	down	Gale	74246	Mus musculus galactose-4-epimerase, UDP (Gale), mRNA.
0.011137	7.19E-04	1.6	down	Dct	13190	Mus musculus dopachrome tautomerase (Dct), mRNA.
0.013198	9.57E-04	1.6	down	Arsa	11883	Mus musculus arylsulfatase A (Arsa), mRNA.
0.012467	8.70E-04	1.6	down	Fam158a	85308	Mus musculus family with sequence similarity 158, member A (Fam158a), mRNA.
9.60E-05	2.60E-07	1.6	down	Ces5	234673	Mus musculus carboxylesterase 5 (Ces5), mRNA.
3.12E-04	2.12E-06	1.6	down	Mocos	68591	Mus musculus molybdenum cofactor sulfurase (Mocos), mRNA.
0.045417	0.006285	1.6	down	C9	12279	Mus musculus complement component 9 (C9), mRNA.

0.006164	2.87E-04	1.6	down	Acy3	71670	Mus musculus aspartoacylase (aminoacylase) 3 (Acy3), mRNA.
0.001971	4.25E-05	1.6	down	Mup5	17844	Mus musculus major urinary protein 5 (Mup5), mRNA.
0.009044	5.27E-04	1.6	down	Cyp2a5	13087	Mus musculus cytochrome P450, family 2, subfamily a, polypeptide 5 (Cyp2a5), mRNA.
0.042515	0.005687	1.6	down	Abcg5	27409	Mus musculus ATP-binding cassette, sub-family G (WHITE), member 5 (Abcg5), mRNA.
0.012302	8.49E-04	1.6	down	Clstn3	232370	Mus musculus calsyntenin 3 (Clstn3), mRNA.
0.001278	2.08E-05	1.6	down	Elovl6	170439	Mus musculus ELOVL family member 6, elongation of long chain fatty acids (yeast) (Elovl6), mRNA.
0.001948	4.15E-05	1.6	down	Sfrs17b	338351	Mus musculus splicing factor, arginine/serine-rich 17b (Sfrs17b), mRNA.
1.60E-04	7.02E-07	1.6	down	Rnf6	74132	Mus musculus ring finger protein (C3H2C3 type) 6 (Rnf6), mRNA.
0.008017	4.41E-04	1.6	down	Gstm4	14865	Mus musculus glutathione S-transferase, mu 4 (Gstm4), mRNA.
0.001529	2.77E-05	1.6	down	Rnpep		
0.004256	1.60E-04	1.5	down	Tiam2	24001	Mus musculus T-cell lymphoma invasion and metastasis 2 (Tiam2), mRNA.
9.38E-04	1.23E-05	1.5	down	Sp5	64406	Mus musculus trans-acting transcription factor 5 (Sp5), mRNA.
0.001864	3.80E-05	1.5	down	Hapln4	330790	Mus musculus hyaluronan and proteoglycan link protein 4 (Hapln4), mRNA.
0.02371	0.002387	1.5	down	4432416J03Rik	78252	Mus musculus RIKEN cDNA 4432416J03 gene (4432416J03Rik), mRNA.
0.02074	0.001934	1.5	down	Pmvk	68603	Mus musculus phosphomevalonate kinase (Pmvk), mRNA.
0.003066	8.91E-05	1.5	down	Nsdhl	18194	Mus musculus NAD(P) dependent steroid dehydrogenase-like (Nsdhl), mRNA.
0.020877	0.001952	1.5	down	Mrps6	121022	Mus musculus mitochondrial ribosomal protein S6 (Mrps6), nuclear gene encoding mitochondrial protein, mRNA.
0.001806	3.53E-05	1.5	down	OTTMUSG00000004551	100038514	PREDICTED: Mus musculus predicted gene, OTTMUSG00000004551 (OTTMUSG00000004551), mRNA.
6.29E-04	6.54E-06	1.5	down	Rnpep	215615	Mus musculus arginyl aminopeptidase (aminopeptidase B) (Rnpep), mRNA.
0.006522	3.14E-04	1.5	down	Sort1	20661	Mus musculus sortilin 1 (Sort1), mRNA.
0.002147	4.84E-05	1.5	down	Uso1	56041	Mus musculus USO1 homolog, vesicle docking protein (yeast) (Uso1), mRNA.
0.002719	7.18E-05	1.5	down	Khk	16548	Mus musculus ketohexokinase (Khk), mRNA.

0.00685	3.47E-04	1.5	down	BC048644	407789	Mus musculus cDNA sequence BC048644 (BC048644), mRNA.
0.003063	8.75E-05	1.5	down	Hspb1	15507	Mus musculus heat shock protein 1 (Hspb1), mRNA.
0.003779	1.31E-04	1.5	down	C77080		
4.95E-04	4.26E-06	1.5	down	Dhrs7b	216820	Mus musculus dehydrogenase/reductase (SDR family) member 7B (Dhrs7b), mRNA.
0.006952	3.54E-04	1.5	down	Fkbp4	14228	Mus musculus FK506 binding protein 4 (Fkbp4), mRNA.
7.95E-04	9.64E-06	1.5	down	Ergic1	67458	Mus musculus endoplasmic reticulum-golgi intermediate compartment (ERGIC) 1 (Ergic1), mRNA.
8.24E-05	1.82E-07	1.5	down	Slc25a37	67712	Mus musculus solute carrier family 25, member 37 (Slc25a37), nuclear gene encoding mitochondrial protein, mRNA.
2.13E-04	1.09E-06	1.5	down	Abcf2	27407	Mus musculus ATP-binding cassette, sub-family F (GCN20), member 2 (Abcf2), nuclear gene encoding mitochondrial protein, mRNA.
0.00283	7.66E-05	1.5	down	Dexi	58239	Mus musculus dexamethasone-induced transcript (Dexi), mRNA.
7.20E-04	8.26E-06	1.5	down	Ecsit	26940	Mus musculus ECSIT homolog (Drosophila) (Ecsit), mRNA.
5.25E-04	5.01E-06	1.5	down	Letm1	56384	Mus musculus leucine zipper-EF-hand containing transmembrane protein 1 (Letm1), nuclear gene encoding mitochondrial protein, mRNA.
0.003901	1.40E-04	1.5	down	Txnl4		
0.007541	4.04E-04	1.5	down	Hyou1	12282	Mus musculus hypoxia up-regulated 1 (Hyou1), mRNA.
0.002661	6.93E-05	1.5	down	Srm	20810	Mus musculus spermidine synthase (Srm), mRNA.
8.32E-05	1.92E-07	1.5	down	Mscp		
0.003311	1.01E-04	1.5	down	6430706D22Rik	381280	Mus musculus RIKEN cDNA 6430706D22 gene (6430706D22Rik), mRNA.
6.54E-04	7.21E-06	1.5	down	Gas7	14457	Mus musculus growth arrest specific 7 (Gas7), mRNA.
0.004472	1.70E-04	1.5	down	Slc6a9	14664	Mus musculus solute carrier family 6 (neurotransmitter transporter, glycine), member 9 (Slc6a9), mRNA.
0.018553	0.001629	1.5	down	Es22	13897	Mus musculus esterase 22 (Es22), mRNA.
0.009842	5.95E-04	1.5	down	Fn3k	63828	Mus musculus fructosamine 3 kinase (Fn3k), transcript variant 1, mRNA.
0.001466	2.58E-05	1.5	down	3000003G13Rik		
7.57E-04	8.88E-06	1.5	down	Dis3l2	208718	Mus musculus DIS3 mitotic control homolog (S. cerevisiae)-like 2 (Dis3l2), mRNA.

0.007741	4.20E-04	1.5	down	Slc1a2	20511	Mus musculus solute carrier family 1 (glial high affinity glutamate transporter), member 2 (Slc1a2), transcript variant 3, mRNA.
0.003901	1.40E-04	1.5	down	AW061290	381110	Mus musculus expressed sequence AW061290 (AW061290), mRNA.
0.023012	0.002275	1.5	down	Pdcd4	18569	Mus musculus programmed cell death 4 (Pdcd4), mRNA.
0.001711	3.31E-05	1.5	down	Coasy	71743	Mus musculus Coenzyme A synthase (Coasy), nuclear gene encoding mitochondrial protein, mRNA.
3.27E-04	2.33E-06	1.5	down	Khk	16548	Mus musculus ketohexokinase (Khk), mRNA.
0.004071	1.49E-04	1.5	down	Acsl5	433256	Mus musculus acyl-CoA synthetase long-chain family member 5 (Acsl5), mRNA.
0.002659	6.92E-05	1.5	down	0610012H03Rik	74088	Mus musculus RIKEN cDNA 0610012H03 gene (0610012H03Rik), mRNA.
0.001455	2.52E-05	1.5	down	Pcca	110821	Mus musculus propionyl-Coenzyme A carboxylase, alpha polypeptide (Pcca), mRNA.
0.031502	0.003653	1.5	down	Abcd3	19299	Mus musculus ATP-binding cassette, sub-family D (ALD), member 3 (Abcd3), mRNA.
0.027191	0.002954	1.5	down	Aqp4	11829	Mus musculus aquaporin 4 (Aqp4), mRNA.
3.08E-04	2.08E-06	1.5	down	Cyb5r3	109754	Mus musculus cytochrome b5 reductase 3 (Cyb5r3), mRNA.
