# Supplementary material for: Pathogenesis of FOLFOX induced sinusoidal obstruction syndrome in a murine chemotherapy model
Source: J Hepatol. 2013 Aug;59(2):318–26. doi: 10.1016/j.jhep.2013.04.014 (PMC3710969; doi:10.1016/j.jhep.2013.04.014)
Supplement: Supplementary Table 6 — Results of qRT-PCR to validate microarray. [file mmc12.pdf]

Entrez ID	Gene Name	Fold Change (FOLFOX vs. Control)	p Value
20201	Mus musculus S100 calcium binding protein A8 (calgranulin A) (S100a8), mRNA.	41	<0.001
17748	Mus musculus metallothionein 1 (Mt1), mRNA.	22	<0.001
	Apolipoprotein A-IV	20	<0.001
20202	Mus musculus S100 calcium binding protein A9 (calgranulin B) (S100a9), mRNA.	79	<0.001
12575	Mus musculus cyclin-dependent kinase inhibitor 1A (P21) (Cdkn1a), mRNA.	21	<0.001
13074	Mus musculus cytochrome P450, family 17, subfamily a, polypeptide 1 (Cyp17a1), mRNA.	9	<0.001
216233	Mus musculus suppressor of cytokine signaling 2 (Socs2), mRNA.	6	<0.001
13118	Mus musculus cytochrome P450, family 4, subfamily a, polypeptide 12B (Cyp4a12b), mRNA.	0.2	<0.001
12686	Mus musculus elongation of very long chain fatty acids (FEN1/Elo2, SUR4/Elo3, yeast)-like 3 (Elovl3), mRNA.	0.2	<0.001
21835	Mus musculus thyroid hormone responsive SPOT14 homolog (Rattus) (Thrsp), mRNA.	0.1	<0.001
277753	Mus musculus cytochrome P450, family 4, subfamily a, polypeptide 12a (Cyp4a12a), mRNA.	0.3	<0.001
13087	Mus musculus cytochrome P450, family 2, subfamily a, polypeptide 5 (Cyp2a5), mRNA.	0.1	<0.001
170439	Mus musculus ELOVL family member 6, elongation of long chain fatty acids (yeast) (Elovl6), mRNA.	0.2	<0.001
55927	Mus musculus hairy and enhancer of split 6 (Drosophila) (Hes6), mRNA.	0.3	<0.001
