# Supplementary material for: Pathogenesis of FOLFOX induced sinusoidal obstruction syndrome in a murine chemotherapy model
Source: J Hepatol. 2013 Aug;59(2):318–26. doi: 10.1016/j.jhep.2013.04.014 (PMC3710969; doi:10.1016/j.jhep.2013.04.014)
Supplement: Supplementary Table 7 — Summary of genes implicated in key enriched pathways. [file mmc13.pdf]

	Gene	Fold Change (FOLFOX vs. Vehicle)
**Cell Cycle Regulation**	*Cdkn1a (p21^{Cip1})*	+ 8.2
	CXCL1	+ 3.3
	GADD45G	+ 2.6
	KLF6	+ 2.3
	STAT3	+ 1.8
	Gas6	+ 1.7
	GADD45B	+ 1.7
	Bax	+ 1.6
	Ccng1	+ 1.5
	Gas7	- 1.5
**Oxidative Stress**	*Mt1*	+ 12.4
	Hmox1	+ 2.4
	Gpx3	+ 2.0
	Mgst3	+ 1.7
	Sod3	+ 1.6
	Gstp1	- 2.3
	Gstm6	- 1.8
	Gstm4	- 1.6
**Angiogenesis**	*Angptl3*	+ 3.3
	Fgf1	+ 2.0
	Ang	+ 1.5
	Flt1	+ 1.5
**Coagulation**	*Cxcl9*	+ 2.1
	Vwf	+ 1.6
**Matrix Remodelling**	*Mmp14*	+ 2.0
	Col6a1	+ 1.9
	Col4a1	+ 1.8
	Col4a2	+ 1.5

Supplementary Table 7. Summary of genes implicated in key enriched pathways
