# Supplementary material for: Pathogenesis of FOLFOX induced sinusoidal obstruction syndrome in a murine chemotherapy model
Source: J Hepatol. 2013 Aug;59(2):318–26. doi: 10.1016/j.jhep.2013.04.014 (PMC3710969; doi:10.1016/j.jhep.2013.04.014)
Supplement: Supplementary Table 8 — The effect of BHA treatment of histological scoring of liver injury. [file mmc14.pdf]

	Control Diet		0.7% BHA	
	**Vehicle (n=5)**	**FOLFOX (n=5)**	**Vehicle (n=5)**	**FOLFOX (n=4)**
**Rubbia-Brandt Grade**				
0	5	0	5	1
1	0	4	0	3
2	0	1	0	0
3	0	0	0	0
**Endothelial Disruption Present**	0	5	0	1*

*p<0.05, Fishers exact test control diet FOLFOX vs. BHA diet FOLFOX

Supplementary Table 8. The effect of BHA treatment of histological scoring of liver injury
